# Decoding cultural conflicts

**DOI:** 10.3389/fpsyg.2023.1166023

**Published:** 2023-09-14

**Authors:** Özgecan Koçak, Phanish Puranam, Afşar Yegin

**Affiliations:** ^1^Goizueta Business School, Organization & Management Area, Emory University, Atlanta, GA, United States; ^2^INSEAD, Singapore, Singapore; ^3^Faculty of Economics, Administrative, and Social Sciences, Department of Business Administration, Kadir Has University, Istanbul, Türkiye

**Keywords:** culture, conflict, vignettes, experiments, Carnegie perspective

## Abstract

As pioneers of the Carnegie Perspective recognized, conflicts in organizations can exist even when incentives of all parties are aligned. These can often be traced to differences in cognitions such as beliefs and values, which are foundational components of any given culture. This paper refines the operationalization of cultural clashes by identifying differences in beliefs about causality (“which actions cause which outcomes”) and morality (in the broad sense of “what is evaluated as desirable”) as two fundamental sources of conflict. In our first study, we demonstrate empirically that participants recognize and distinguish between these two sources of conflict. In our second study, we test the hypotheses that while misalignments in either causal or moral codes increase observers' perceptions of relationship conflict, negative affect, likelihood of avoidance, and lower perceived likelihood of conflict resolution, the effects are stronger for misalignments in moral codes than misalignments in causal codes and strongest when both causal and moral codes are misaligned. We test these arguments using vignette-based experimental studies. Our findings support our hypotheses. This research has significant implications for the understanding of conflict dynamics within and beyond organizational contexts. By recognizing the pivotal role of cultural differences in shaping conflicts, organizations and decision-makers can better anticipate, manage, and potentially preempt such conflicts.

## 1. Introduction

Central to *A Behavioral Theory of the Firm* (Cyert and March, 1963) is the idea that organizations cannot be treated as unitary entities with a single goal. When an organization's members have different goals, conflict naturally ensues. Moreover, the Carnegie perspective highlights that differences in information, attention, and problem representation can also be consequential for conflict, even if there is alignment on ultimate goals (Simon, 1947; March and Simon, 1958). For instance, in an early articulation of this idea, Dearborn and Simon (1958) documented how structural differentiation within a company may lead executives in different units to reach different and ultimately conflicting interpretations of the same business situation.

Yet, the importance of cognition-driven sources of conflict appears to have disappeared from the agenda of behavioral theories of the firm. Concluding a recent survey of the extensive literature on information processing and organization design, which is to a large extent inspired by the Carnegie perspective, Joseph and Gaba (2020) noted that: “… the literature largely overlooks the potential for conflict in decision-making. This shortcoming reflects, inter alia, the belief that conflict results from divergent interests and poor incentive design (Gibbons, 2003).” We believe this lacuna points to an emergent division of labor between organization science and organizational economics, in which the latter is presumed to be adequately covering conflict through its focus on problems of misaligned interests between principals and their agents, leaving the former free to pursue other topics. However, as Joseph and Gaba (2020) point out, such a division of labor rests on the faulty premise that conflicts result only from imperfect incentive design. Incentives are rewards (such as payments, career progression, or benefits) that individuals (expect to) get out of certain outcomes, and they divide value between the principal and agents (Lazear, 2018). Poorly designed incentives are an important source of conflict within organizations, both among peers and between superior and subordinates (Gibbons and Roberts, 2013), but they are by no means the only source of conflict.

In parallel, research on organizational culture has progressed largely independent of the behavioral theories of decision-making and learning that Joseph and Gaba (2020) reviewed and has developed a substantial body of theory and a repertoire of tools that are relevant to studying cognition-driven conflicts in organizations. Like psychological studies of national cultures and sociological studies of social groups, studies of organizational cultures conceptualize “culture” most basically as shared cognitive constructs such as values, beliefs, and norms (Chatman and O'Reilly, 2016). In this view, different organizations within the same country can have distinct organizational cultures. This is because organizational cultures, as shared cognitions, evolve as a learned response to organizational problems. This idea is reflected in Schein's definition of culture as: “(1) A pattern of shared basic assumptions, (2) invented, discovered, or developed by a given group, (3) as it learns to cope with its problems of external adaptation and internal integration, (4) that has worked well-enough to be considered valid and, therefore (5) is to be taught to new members as the (6) correct way to perceive, think, and feel in relation to those problems.” (Schein, 2010, 2012, p.313).

However, the *extent* to which cognitions are shared within an organization—what is referred to as the “strength” of a culture (Chatman and O'Reilly, 2016; Marchetti and Puranam, 2022)—can vary significantly. Furthermore, different sub-cultures can exist in the same organization, leading to divergent interpretations and strategies for action (e.g., Howard-Grenville, 2006). The idea that a group can have a weak culture or that it might contain sub-groups with different cultures is central to organizational studies adopting the “culture as toolkit” view of culture from sociology, which studies how agents can strategically exploit such variability (Swidler, 1986; Giorgi et al., 2015). It is also a central assumption in the literature on moral reframing within psychology, which studies how mediators can create support for polarizing issues across sub-cultures by bridging differences in beliefs and values (Feinberg and Willer, 2019).

In any setting (within or outside organizations), individuals might disagree about the core tenets of an issue because they belong to different groups with distinctive cultures (e.g., sub-units of an organization or different tribes in a nation) or because the group that they both belong has a weak culture. Thus, sub-cultural and intra-cultural variation in organizations is an important source of potential conflict in organizations, even if individuals have the same incentives. Cognitive conflicts ultimately involve differences in cognitions *between* people (and between groups of people) and research on culture gives us access to a powerful set of ideas about the nature and stability of differences in beliefs and values among people. We do not claim that culture is the only source of such differences but rather that it is a sufficiently important one.

In this study, we attempt to extend and refine the idea of cognition-driven conflicts through three contributions. First, we link the problem of cognition-driven conflict in organizations to cultural clashes. This broadens (beyond incentive misalignment) the notion of conflict in organizational settings, which was salient to pioneers of the Carnegie perspective, but which has since receded in importance in research within this perspective (Joseph and Gaba, 2020). Second, we refine the operationalization of cultural clashes by identifying differences in beliefs about causality (“which actions cause which outcomes”) and morality (in the broad sense of “what is evaluated as desirable”) as two fundamental sources of conflict. In doing this, we draw on the construct of cultural codes—defined as fuzzy mappings between distinct types of cognitive constructs (Koçak and Puranam, 2023). In our first study, we demonstrate empirically that participants recognize and distinguish between these two sources of conflict based on differences in cognitions pertaining to causality or morality. Third, we build on research on inter-personal conflict in teams, attitude polarization, and moral conviction to propose that conflicts whose roots lie in differences in causal codes are perceived by third parties as easier to resolve than conflicts that arise from differences in moral codes. In our second study, we test the hypotheses that while misalignments in either causal or moral codes increase observers' perceptions of relationship conflict, negative affect, likelihood of avoidance, and lower perceived likelihood of conflict resolution, the effects are stronger for misalignments in moral codes than misalignments in causal codes. We end with a discussion of implications for organizations and potential interventions to forestall or resolve conflicts.

## 2. Micro-foundations of cultural clashes

Insights about cultural clashes come to us from at least three different bodies of literature—on culture and cognition, interpersonal conflict in teams, and attitude moralization and polarization. In what follows, we first review the relevant literature. Next, we build on and extend the literature on culture and cognition to develop the notion of a “chain of reasons” that capture the cognitive underpinnings of behavior and its justification. We then use the literature on attitude polarization and team conflict to theorize about the different effects of beliefs and attributions about links in the chain that are concerned with causality vs. links pertaining to morality.

### 2.1. Related literature

Culture clash exists when interacting individuals do not share one or more cultural cognitions. Studies show that clashes can give rise to failures of communication and coordination, and even outright conflict, especially in task groups with members separated by occupational histories or geography (e.g., Bechky, 2003; Carlile, 2004). Representational gaps (“rGaps”)—inconsistencies between individuals' definitions of a team's problem—limit knowledge integration and increase the likelihood of conflict (Cronin and Weingart, 2007, 2019). Not all differences in assumptions, values, or beliefs need to be detrimental, however. For instance, the diversity of cognitive styles and views is thought to spur innovation (Corritore et al., 2020).

Research on interpersonal conflict in work groups also focuses on differences in beliefs and values and can therefore be treated as pertaining to cultural clashes. This research suggests that the *content* of disagreement leads to different types of conflict, some of which are more detrimental than others for team performance. Four types of inter-personal conflict have received the most attention: task, process, relationship, and status (see Greer and Dannals, 2017, for a review). Task conflict stems from disagreements about “the content of the tasks being performed, including differences in viewpoints, ideas, and opinions” (Jehn, 1995, p. 258). “Task-related debates can be about either the content or the process of the task. Task content is about what to do (e.g., a new marketing campaign), in contrast to task process, which is about how to do it (e.g., delegation of responsibilities)” (Jehn et al., 1999, p. 743). The latter is often separated from the former and referred to as process conflict (Jehn, 1995; Jehn et al., 1999). Relationship conflict refers to “conflict over workgroup members' personal preferences or disagreements about interpersonal interactions, typically about non-work issues such as gossip, social events, or religious preferences (Jehn, 1995, 1997).” (Jehn et al., 1999, p. 745). Status conflict refers to disagreements over relative status positions in a team's social hierarchy (Bendersky and Hays, 2012). Recently, Brown et al. (2022) have added ethical conflicts—stemming from disagreements about moral convictions and normative conventions—as a fifth type of workplace conflict.

Note that relationship conflict is different from the other types of conflict in that it does not (only) refer to the content of disagreement but also to conflict attitudes and behaviors—to there being “tension, animosity, and annoyance among members within a group” (Jehn, 1995, p. 258), i.e., to disagreements being “hot.” This is important to note because empirical studies find that task conflict can have a positive impact on group performance when it does not co-occur with relationship conflict (De Wit et al., 2012). Conversely, an inductive study of conflict-resolution tactics used by autonomous work groups (study groups) finds that successful teams share a tendency to focus on content rather than style (Behfar et al., 2008). Another study finds that groups that can use coping strategies to decouple task conflict from relationship conflict are more likely to benefit from it (Pluut and Curşeu, 2013).

While informative, the prior literature leaves open two issues that are crucial to progress on our research agenda.

First, a relevant question is whether disagreements rooted in particular content lead to affective reactions and relationship conflict. On the one hand, it is possible that the content of cognition is unrelated to whether disagreements generate relational or emotional conflict. Research on team conflict suggests that presumably, disagreements over any topic (including ethical, status, process, or task issues) can all turn “hot.” For instance, Brown and colleagues find that task or ethical conflicts have the same propensity to create or co-exist with relationship conflict (Brown et al., 2022, p. 1135). Others find that the likelihood of task conflicts to develop into relationship conflicts depends on factors such as intergroup trust (Simons and Peterson, 2000) and coping strategies (Behfar et al., 2008; Pluut and Curşeu, 2013). Similarly, research on attitude polarization, which identifies antecedents of emotionally charged attitude conflicts characterized by parties' intolerance of each other's positions (Minson and Dorison, 2022) does not mention the content of cognitions at all. Rather, it focuses on three antecedents: outcome importance, actor interdependence, and evidentiary skew (parties' belief that the weight of evidence overwhelmingly supports their respective points of view).

On the other hand, some studies suggest that content and emotion are not entirely divorced. Research on moral conviction shows that individuals' *perception that some decisions, choices, judgments, and attitudes are moral* leads to conflict when there is disagreement on those attitudes (Skitka et al., 2021). People who feel their preferences to be motivated by moral commitments are less tolerant of others with dissimilar preferences and avoid interacting with them (Skitka et al., 2015). While suggestive, the moral conviction literature does not fully explore the link between content of disagreements and the negative affect and relationship conflict that might follow. For instance, Skitka et al. (2021, p. 350) emphasize that “morality is not an essential feature of some decisions, choices, judgments, or attitude domains—rather, it is a meta-perception people have about some of their decisions, choices, judgments, and attitudes that can vary in strength.” Instead, the focus of this literature has been on the range of application of beliefs. Moral beliefs are assumed to be universally applicable, and thus distinguished from preferences (held by individuals) and normative conventions (recognized as being specific to particular social groups). It is this belief in universality that, when violated by perceptions of difference, leads to moral conflict. In other words, while “the moral significance people attach to different issues varies over time, cultures, and individuals,” issues that are seen as morally significant—and thus distinguished from preferences and conventions—are tied to emotions, resist change, and create intolerance for differing viewpoints. That said, studies in this line of research do not examine whether certain types of cognitions (across a range of issues) might more or less likely be perceived as morally significant (across cultures).

A second shortcoming we perceive is that neither the literature on conflict nor the literature on moralization explicitly examines differences in causal reasoning. The literature on managerial cognition, in contrast, is overwhelmingly about causal understandings (Walsh, 1995). Methods used for strategy formulation also focus on clearly mapping cause–effect relationships (Carroll and S*ϕ*rensen, 2021), suggesting that strategic decision-making requires an explicit focus on cognitions about causality.

While “task conflict” in the team conflict literature comes close to finding sources of conflict in disagreements about cause–effect relationships, it is much broader in that it can include disagreements on what the team's task is and what the goals of the team are. For instance, the task conflict sub-scale within the intragroup conflict scale uses items such as “How frequently are there conflicts about ideas in your work unit?” and “How often do people in your work unit disagree about opinions?” (Jehn et al., 1999). Meanwhile, “process conflict” refers to the team's understanding of how the task can be accomplished, but is too narrow, in that it refers to how the task is to be accomplished by the team, through division of labor. The sub-scale consists of three questions: “How often do members of your work unit disagree about who should do what?,” “How frequently do members of your work unit disagree about the way to complete a group task?,” and “How much conflict is there about delegation of tasks within your work unit?” (Jehn et al., 1999). Thus, neither scale focuses on the cause–effect relationships as being the source of contention. If a conflict arose from differences in beliefs about causality—for instance, the effectiveness of particular tools or materials for building a product, or whether a proposed initiative will contribute to employees' felt inclusion—both the task conflict and the process conflict scales might pick it up but neither would be able to distinguish it from differences in how much individuals value the various actions or outcomes—such as whether the team should place greater value on the effectiveness of tools or their impact on the environment or whether felt inclusion or demographic diversity should be a goal of the team.

In what follows, we address these two shortcomings by considering the cognitive underpinnings of such disagreements. We propose a typology of cognitions about causality and desirability that in combination motivate preferences and behavior and, when they differ, can lead to disagreements.

### 2.2. Causal and moral codes in a chain of reasons that underpin behavior

Within behavioral strategy, representations play a central conceptual role in explaining strategic reasoning and choice (e.g., Gavetti and Levinthal, 2000; Gavetti and Rivkin, 2007; Levinthal, 2011; Csaszar and Levinthal, 2016; Puranam and Swamy, 2016; Csaszar, 2018). Most often, the term refers to individual decision-makers' understanding of their task environment, connecting potential actions to their expected payoffs. However, this umbrella term can encompass a wide range of cognitions. In this study, we focus on two types of cognitions that are relevant for decision-making in organizations: desirability of outcomes and ways to achieve outcomes.

Following Koçak and Puranam (2023), we express these two cognitions as codes. The construct of a “code” builds on that of “schema”—as networks of connected cognitive elements that store cultural knowledge and guide action (DiMaggio, 1997; Strauss and Quinn, 1997; Hunzaker and Valentino, 2019; Cerulo et al., 2021). As with schema, a code specifies a *mapping* between concepts, where the strength of mapping is adjusted through experience. Unlike schema, a code specifies the type of concepts that are joined and implies a directional tie (e.g., mapping cause to consequence). When codes shape an individual's behavior, we say they are *using* a code. Individuals can also have *expectations* about the codes others use.

“Causal codes” are beliefs about how the world works, expressed as (fuzzy) mappings between causes and effects. Similar concepts have been used in research on managerial cognition, referred to variously as “cause maps” (Bougon et al., 1977), “beliefs about causes and effects” (Ford and Hegarty, 1984), and “causal beliefs” (Porac et al., 1989). *Using* a causal code (e.g., about how new technology affects the emissions from a production process), an agent can choose or advocate for a particular action (e.g., to adopt the technology). *Expecting* another agent to use a particular causal code, an agent might tacitly align their actions to it (e.g., only suggest the new technology to leaders who believe it to be effective).

By “moral codes,” we are referring to evaluations of entities, actions, or outcomes as desirable or undesirable, again expressed as a (fuzzy) mapping from the former to the latter. We construe these broadly, to include desirability attached to any outcome that is relevant to organizational behavior (including profitability), and not only pro-social outcomes (such as social impact).^1^^,^^2^ *Using* a moral code (e.g., about whether reducing emissions beyond the legally mandated limit is a moral duty), an agent can defend an action (e.g., adopting the technology despite its high costs). *Expecting* a moral code to be used by their leaders, an agent can advocate for a particular action (e.g., not adopting the technology) even if it conflicts with their own moral code.

**Figure 1:**
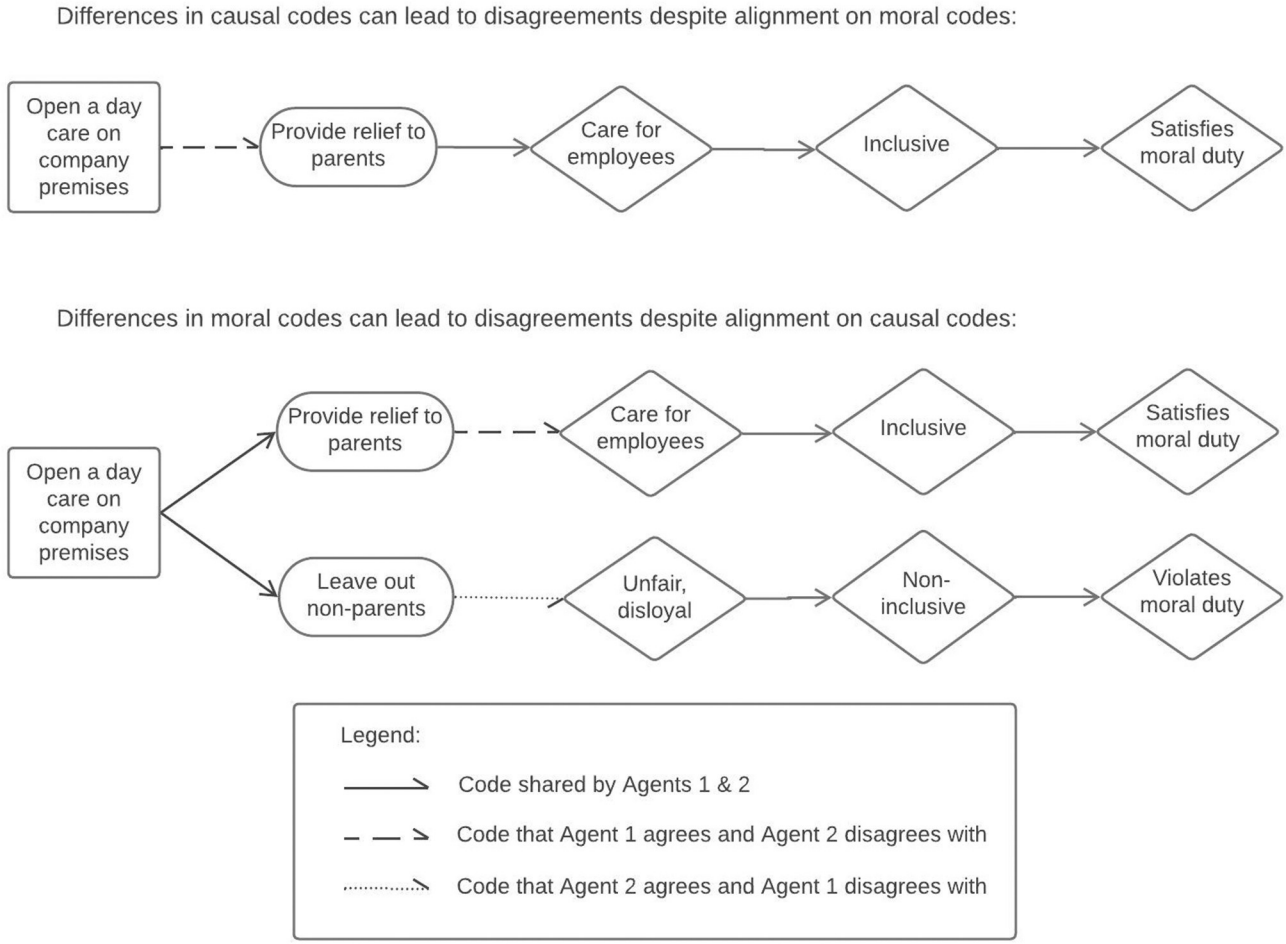
Schematic representation of disagreements that arise from differences in causal or moral codes.

In Figure 1, we illustrate causal and moral codes concerning another hypothetical issue—the opening of a daycare center in a company. The causal code refers to whether opening a daycare center on company premises would provide relief to employees with children. The moral code refers to whether providing relief to parents would support the positively valued dimension of inclusivity (because it demonstrates care) or if it would be non-inclusive because it leaves out employees without children (and is therefore unfair). A combination of codes such as this depicts how decisions could be motivated or rationalized with a “logic of consequences” (March and Olsen, 2011), under the assumption that any goal-directed behavior requires people to have an understanding of what outcome they want (specified in a moral code) and also of how to reach that outcome (specified in a causal code).^3^

Note that this schematic representation does not necessarily show how people might describe their own reasoning. We do not assume people to expressly articulate the codes that motivate their actions or to separate them into causal and moral codes. Nor do we assume that people can consciously access the codes that drive their own behaviors. People have a broad set of codes, only a part of which is activated at any given time. In any given situation, codes may be activated automatically without conscious deliberation or with deliberation. The chain of reasons may remain tacit and unarticulated until agents are asked to explain their behavior. And even then, people may not be able to accurately pinpoint what had driven their behavior or preferences. As a result, codes that motivate action need not be the same as the codes that are used to justify action.

Both causal and moral codes can be acquired through personal experiences or socially transmitted between people. In either case, because experiences that shape codes are likely to vary across groups and because transmission of codes is more likely within than across groups, codes are more likely to be similar (but not identical) within groups and different across groups. It is in this sense that individual cognition is “cultural” and groups have distinct cultures (Strauss and Quinn, 1997). Thus, while we focus on conflicts that arise from differences in individuals' codes, these differences are ultimately reflections of differences within and between (sub)-cultures.

The key premise of our argument is that differences in either causal or moral codes can lead to disagreements on preferred courses of action. In the top panel in Figure 1, a difference in opinion arises from differences in causal codes, as one agent believes that a daycare center on company premises would support parents while the other agent does not. In the lower panel, the disagreement arises from differences in moral codes: one agent believes that supporting only employees with children would be unfair, while the other one does not.

Fuzziness in codes (i.e., the mapping between concepts being one-to-many, many-to-one, or many-to-many) can also create disagreements. In the lower panel of Figure 1, both agents believe that daycare centers both provide relief to parents and leave out non-parents. Situational cues or particular ways of framing the debate may focus agents on the first belief while others focus them on the second belief. Thus, even with very similar codes, fuzziness in codes can, in some situations, create disagreements between these two agents.

In this study, we focus on a potential observer's perspective, corresponding to the viewpoint of a potential mediator of conflicts. Third parties observing other agents' disagreements may perceive or analyze these in terms of causal and moral codes. In doing so, they are likely to rely on their *expectations* about the codes that others have and use. For instance, a third agent, who expects that peoples' beliefs about the consequences of daycare centers for parents' welfare will vary, may accurately diagnose the source of disagreement depicted in the top panel of Figure 1 as arising from differences in causal codes. Conversely, an observer who does not recognize the possibility for this variability or uncertainty in causal codes might erroneously assume that the disagreement stems from a difference in moral codes.

### 2.3. Effects of perceived misalignments in causal and moral codes

Whether or not they are accurate (i.e., correspond to the codes that motivated agents' behavior), the way agents diagnose the root causes of a conflict is likely to impact the actions they take and therefore the likelihood of conflict resolution. Therefore, the effects of third-party mediation of conflicts should depend on how this party diagnoses the root cause of cultural conflict.

We propose that conflicts that are traced to misalignments in causal codes will appear to be easier to resolve than moral codes, in turn generating attitudes and behaviors that increase the likelihood of conflict resolution. There are several reasons to think so.

People may intuitively understand that cause–effect relationships lend themselves to evidence-based reasoning and debate, while moral codes do not. Knowing that cause–effect relationships can lend themselves to evidence-based resolution, individuals can hold off moralizing differences of opinion. Even in the absence of required evidence, this can make way for reasoned debate and easier resolution by preventing relationship conflicts, negative affect, and avoidance behaviors.

Conversely, attributions of misalignments in moral codes can lead to relationship conflict, negative affect, and avoidance behaviors (Jehn, 1995; Behfar et al., 2008; Pluut and Curşeu, 2013). This would close off avenues for resolution through debate. Research on moral conviction shows that this might happen because moral codes are assumed to be universally applicable and any argument that they are not, any encounter with people who contest this universality may be perceived as an affront to the way the world is supposed to be (Skitka et al., 2021). Perceptions of misalignments in moral codes can make resolution less likely also if these (more than causal code differences) are associated with any of the three antecedents that the attitude polarization literature identifies as increasing likelihood of conflict: outcome importance, actor interdependence, and evidentiary skew (parties' belief that the weight of evidence overwhelmingly supports their respective points of view) (Minson and Dorison, 2022). Finally, it might be possible that differences in moral codes (which are associated with emotions) generate negative emotion because people want to be aligned in their emotional responses toward issues. That is, we want to feel positive or negative affect toward the same objects and failure to do so creates barriers to convergence.

Thus, we hypothesize:

Hypothesis 1. Perceived misalignment of either causal or moral codes decreases perceived likelihood of reaching an agreement.
Hypothesis 2. Perceived misalignment of moral codes decreases perceived likelihood of reaching an agreement to a greater extent than misalignment of causal codes.
Hypothesis 3. Perceived misalignment in moral codes amplifies the effect of causal codes on perceived likelihood of reaching an agreement.

## 3. Study 1

The study's purpose was 2-fold; to develop an instrument that allows us to measure attributions of sources of conflict to misalignments in causal and/or moral codes and to test if individuals distinguish between causal and moral codes. We generated scale items that reflect our conceptualization of causal codes as pertaining to cause–effect relationships between actions and their consequences and of moral codes as assigning desirability to actions or their consequences. We then tested whether study participants can reliably use these items to diagnose the source of disagreement in vignettes presenting a fictional debate between two managers about their organization opening a daycare center for the children of employees. Although we had not designed Study 1 to test our hypotheses, we also report exploratory tests of H2.

### 3.1. Participants

We recruited participants from the USA using the Prolific.co platform. Prolific.co is an online platform similar to Amazon Mturk (Buhrmester et al., 2018; Aguinis et al., 2021) that allows researchers to recruit participants for online studies. It has been shown to yield data quality comparable to Amazon Mturk with lower participant dishonesty and higher naiveté (Peer et al., 2017). Our target sample size was 100 participants (Hair et al., 2010). A total of 107 participants attempted the survey, of which seven left before completion. In addition, we excluded data from five participants whose response to the comprehension check question was not accurate. The final sample of 95 participants ranged between the ages of 18 and 66 years (*M* = 32.65, *SD* = 11.31) and predominantly identified as white (*n* = 71), followed by “Other” (*n* = 14), African American (*n* = 7), and Hispanic (*n* = 3).

Given the content of the vignette, we also included questions about whether participants had children and if daycare services were available to the participants at their place of employment. Most participants (*n* = 74) did not have children. Of those with children, none had access to daycare on company premises. Finally, participants responded to two questions inquiring about their political orientation on social and economic issues using an 11-point response scale (1-strongly liberal/left-wing, 11-strongly conservative/right-wing). The items had good reliability using the Spearman–Brown coefficient (*r* = 0.894), allowing us to create a single political orientation measure. The majority of our participants self-identified on the left of moderate (*n* = 70) with 21% (*n* = 20) indicating that they were strongly liberal (picking the left-most point on the scale). A minority indicated that they were either moderate (*n* = 12) or right-wing (*n* = 13).

### 3.2. Procedure

The study used a vignette design. After reading and accepting the informed consent form, participants were presented with a brief introduction, which indicated that they would read a conversation between two HR managers at a mid-sized company. The managers were discussing an employee suggestion to open a daycare center for employees' children at their workplace. This introduction was identical for all participants. Thereafter, participants were randomly assigned to one of two conditions (misalignments based on moral codes or causal codes) and viewed slightly different versions of a brief conversation. Specifically, the content of the arguments presented by the HR managers differed across conditions. The full text of the conversation is presented below. Italics indicate causal condition arguments. In the causal code misalignment condition, both parties relied on the consequences of a daycare center to support their position. In the moral code misalignment condition, they emphasized the moral obligations associated with opening a daycare center.

Wilson: We should open a day care center on company premises, for employees' kids.

Smith: I think that's a bad idea.

Wilson: *Opening a day care center might reduce absenteeism and thus help the bottomline./*This is the right thing to do. We say we are a family, we should act like one.

Smith: *But it opens the company to legal liability around running a childcare center./*I don't think it's fair to use company funds for a project that will only benefit some of the employees.

After reading the vignette, participants responded to an open-ended question about the root cause of the disagreement (“Why do you think Wilson and Smith disagree about opening a daycare center at their workplace? What is the root cause of their disagreement?”) and a multiple-choice question about the likelihood of conflict resolution (“How likely do you think it is that Wilson and Smith can reach an agreement?”). They were then presented with two versions of the instrument, one distal and abstract and the other proximate and concrete. Sample items from the distal instrument include “They disagree about the consequences of their respective proposed actions” and “They disagree because they have conflicting values.” Sample items from the proximate instrument include “They disagree because they expect different consequences to follow from a company-owned day care center” and “They disagree about whether it is morally acceptable for a company to offer day care for its employees' kids” (see Appendix 1 for a list of all items). Participants assessed each statement using a 5-point Likert response scale (1-strongly agree, 5-strongly disagree). The scale scores were reversed during the analysis such that higher scores indicated higher perceived misalignment in codes. This question block was followed by the intragroup conflict scale, also evaluated on a 5-point Likert response scale (1-strongly agree, 5-strongly disagree) (Jehn and Mannix, 2001). The questionnaire concluded after participants provided brief demographic information.

### 3.3. Analyses and results

**Figure 2:**
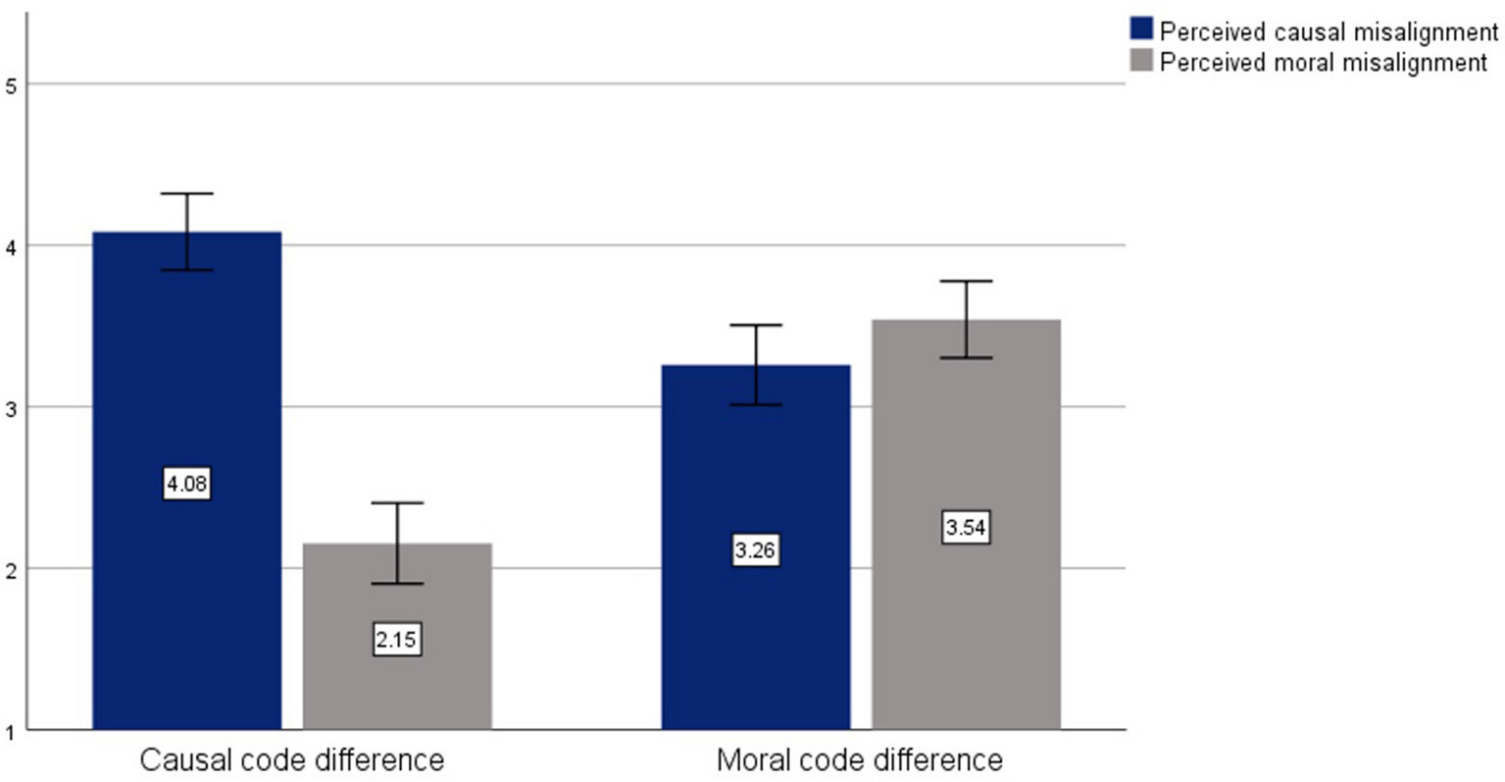
Study 1 results: perceived code misalignment by condition (distal scale).

We report analyses here on the distal scale, which we subsequently use to check our manipulations in Study 2 (see Appendix 2 for analyses on the proximate scale, which yield the same pattern of results). All analyses of the code misalignment instrument were conducted on Jamovi 2.2 (The jamovi project, 2022). Confirmatory factor analysis supported a two-factor structure [Comparative Fit Index (CFI) = 0.998, Root Mean Square Error of Approximation (RMSEA) = 0.017] by common acceptance levels (Bentler, 1990; Hu and Bentler, 1998; Ullman, 2006) (see Appendix 1 for further details, as well as an exploratory factor analysis and additional validation with an independent sample). Moreover, the sub-scales exhibited good reliability (*α*_causal_ = 0.820, *α*_moral_ = 0.872). Thus, we calculated mean moral code misalignment and causal code misalignment scores to be used for the second part of the analysis, which we display by condition in Figure 2.

To assess whether participants were able to identify sources of disagreement in misalignment of moral and causal codes, we conducted *t*-tests of attributions across conditions. Participants in the causal code difference condition attributed the disagreements to misalignments in causal codes significantly more (*M* = 4.08, *SD* = 0.73) than participants in the moral code misalignment condition (*M* = 3.26, *SD* = 0.92), *t*_(93)_ = 4.66, *p* < 0.001. In contrast, participants in the moral code difference condition showed a significantly higher tendency to attribute disagreements to misalignments in moral codes (*M* = 3.54, *SD* = 0.89) compared to participants in the causal code difference condition (*M* = 2.15, *SD* = 0.77), *t*_(93)_ = −0.790, *p* < 0.001. These findings demonstrate that individuals were able to reliably distinguish between moral code misalignments and causal code misalignments.

However, we also observe some spillover effects: even when we manipulated the vignette to indicate that the managers disagreed on moral codes, participants' attributions to causal code misalignments were almost as high as attributions to moral codes. Comparing the scale scores *within* each condition, we find that the difference between moral and causal code misalignment scores was significant in the causal code condition, Δ = 1.93, *t*_(38)_ = 12.338, *p* < 0.001, but only marginally significant in the moral code condition, Δ = 0.28, *t*_(55)_ = −1.715, *p* = 0.092.

Finally, while our main concern in this study was not hypothesis testing, we expected, per H2, that participants' perceptions of ease of conflict resolution would vary across conditions. A one-way ANOVA revealed that there was not a statistically significant difference in the perceived likelihood of conflict resolution between the two conditions [F_(1,93)_ = 0.006, *p* = 0.936]. Thus, initial evidence suggests that both types of conflicts are perceived to be equally difficult to resolve, in contrast to H2.

## 4. Study 2

Study 2 tests Hypotheses 1 through 3, using the instrument developed in Study 1 to check the effectiveness of our manipulations. In this study, we used two vignettes, one concerning the same daycare problem used in Study 1 and a second one concerning investment in technology to reduce greenhouse emissions. We employed a 2 (vignette) × 2 (moral code aligned/misaligned) × 2 (causal code aligned/misaligned) experimental design. The vignette was a within-subject factor; each of the moral code and causal code misalignment was a between-subject factor. Rather than asking participants to infer sources of disagreements from conversations, the vignettes stated whether two managers in an organization disagreed due to misalignments in causal or moral codes and asked for predictions about the likelihood that the managers would be able to resolve their conflict. Notably, the vignettes did not mention “culture” or whether others in the organization shared the managers' opinions.

In our first attempt at Study 2 (which we will henceforth call Study 2a, preregistered at https://doi.org/10.17605/OSF.IO/T28WE), we combined three items to measure the outcome variable (likelihood of conflict resolution): perceived difficulty of reaching an agreement, extent of conflict experienced, and desirability of future collaboration. Using this DV, we found support for Hypothesis 1 in a sample of 463 participants. Tests for Hypothesis 2 were insignificant when the items were combined (*p* = 0.55). In *post-hoc* models separately examining the three items of the outcome measure, we found a misalignment in moral codes to have a marginally stronger effect on the desire to collaborate in the future (*p* = 0.09). However, misalignment in causal codes had a stronger effect on perceived difficulty of reaching an agreement and there was no difference between misalignment in the two codes on experience of conflict. We had not registered Hypothesis 3 for this experiment, but we did find misalignment in the two codes together to have a greater effect on all three items relative to misalignment in causal codes alone. We present the full set of results in Appendix 3.

Given the inconclusive results in tests of H2, and realizing that the three outcome items may tap into different dimensions of the overall outcome measure, we designed Study 2b (preregistered at https://doi.org/10.17605/OSF.IO/VZ6NA), using the same factorial design as Study 2a but decomposing the outcome into four sub-categories and measuring each with multiple items. Our hypotheses, revised to account for the finer grained decomposition of the outcome variable (perceived likelihood of reaching an agreement), are as follows:

Hypothesis 1. Perceived misalignment of either causal or moral codes (i) decreases the perceived likelihood of reaching an agreement on the current problem, (ii) increases the perception of relationship conflict, (iii) increases the perceived likelihood of parties avoiding (vs. engaging with) each other in the future, and (iv) increases perceptions of negative affect developing between the two parties.
Hypothesis 2. Perceived misalignment of moral codes (i) decreases the perceived likelihood of reaching an agreement on the current problem, (ii) increases the perception of relationship conflict, (iii) increases the perceived likelihood of parties avoiding (vs. engaging with) each other in the future, and (iv) increases perceptions of negative affect developing between the two parties to a greater extent than misalignment of causal codes.
Hypothesis 3. Perceived misalignment in moral codes amplifies the effect of causal codes on (i) the perceived likelihood of reaching an agreement on the current problem, (ii) the perception of relationship conflict, (iii) the perceived likelihood of parties avoiding (vs. engaging with) each other in the future, and (iv) perceptions of negative affect developing between the two parties.

We report the results of hypothesis tests using data from Study 2b below.

In addition to hypothesis tests, we explore whether people might be more likely to attribute disagreements to moral or causal codes in the absence of any information about (mis)alignment in their codes. To do this, we included a “no information” condition in addition to the experimental conditions in Study 2b, in which we state that there is a disagreement but do not state whether these stem from disagreements on moral or causal codes. Moral codes receive greater coverage than causal codes in the literature on conflict, which suggests that people may generally (and especially when there is limited information about the sources of disagreement) be more likely to attribute conflicts to misalignments in moral codes than to causal codes. This might arise because prevailing lay theories of conflict may see conflicting interests (rather than differences in perception or information) as the primary source of collaboration failure. We also suspect, however, that the degree to which a disagreement is assumed to arise from causal or moral code misalignments varies by (culturally specific) priors across topics and we might therefore find differences in attributions across the two vignettes.

Finally, we also report exploratory analyses on responses to an open-ended question we included in Study 2a, asking participants to recommend interventions that might increase likelihood of agreement.

### 4.1. Participants

We recruited participants in the USA using the Prolific.co platform. We paid all participants a fixed compensation (5 USD). A total of 502 participants completed the survey. We discarded 27 responses where the participant had failed either of two attention check questions, leaving a final sample of 475 participants. The sample ranged between the ages of 18 and 83 years (*M* = 36.73, *SD* = 13.42) and predominantly identified as white (*n* = 330). There were 235 male and 230 female participants, the remaining identified as non-binary (*n* = 10). In response to a question asking about the level at which they received science education, 37.7% (*n* = 179) reported they had scientific training at or below the high school level, 56.6% (*n* = 269) at the college level, and 5.7% (*n* = 27) of the participants indicated they had studied science in graduate school. Only 14 participants worked in an organization that offered childcare services. An additional 22 participants received childcare support from their employer. Majority of our participants (*n* = 289) considered climate change to be a global emergency and believed that the world should urgently do everything necessary to combat it. Only 40 participants did not consider climate change to be an emergency.

### 4.2. Materials and procedure

The study employed a 2 (order of vignettes) × 2 (causal code misalignment) × 2 (moral code misalignment) fully crossed repeated measures design. The order of vignettes was a between-subjects factor. We do not find order effects and therefore do not report them. The source of disagreement (causal and/or moral) was a within-subjects factor and was randomly assigned for each vignette. This created four conditions, that we refer to as C(m)M(m) (misalignments in both causal and moral codes), C(m)M(a) (misalignment only in causal codes), C(a)M(m) (misalignment only in moral codes), and C(a)M(a) (no misalignments in either causal or moral codes). We also included a “no information” condition for both vignettes where no information was given on the source of disagreement.

After participants read and accepted the consent form, they were informed that they would read two workplace scenarios concerning two different sets of mid-level managers. Both vignettes indicated that the managers were working for a mid-sized company and had been asked to consider a proposed initiative. In one vignette, the proposal concerned opening a daycare facility for employees' children. In the second, the managers were to evaluate a carbon emission reduction technology that might reduce emissions below the legal threshold, which the company was already meeting. In both cases, the text presented participants with the private and independent thoughts and opinions of each manager, which served as our manipulation. Table 1 presents the manipulations for each condition and each vignette.

**Table 1:** Study 2—conditions and vignettes.

**Condition**	**Vignette: green technology**	**Vignette: daycare**
C(a)M(m) Causal codes aligned, moral codes misaligned	In thinking independently and privately about this proposal, Price and Powell **agreed** that the project would **yield substantial carbon emission reductions**, bringing total emissions **far below the legally required threshold**. However, they also had **different views about the moral implications** of the project. **Price thought it was a moral duty** for the company to do as much as it can for the environment, including reducing emissions below what is required by law but **Powell did not**.	In thinking independently and privately about this proposal, Smith and Wilson **agreed that an on-site daycare facility would serve to provide relief to parents**. However, they also had **different views about the moral implications** of the project. **Smith thought it was a moral duty** for the company to do something to help parents better manage work-life balance but **Wilson did not**.
C(m)M(a) Causal codes misaligned, moral codes aligned	In thinking independently and privately about this proposal, Price and Powell **disagreed** on whether the project would yield **substantial carbon emission reductions**, bringing total emissions **far below the legally required threshold**. Price thought it would but Powell did not. However, they **both thought it was a moral duty** for the company to do as much as it can for the environment, including surpassing the legal emissions threshold.	In thinking independently and privately about this proposal, **Smith and Wilson disagreed on whether an on-site daycare facility would serve to provide relief to parents**. Smith thought it would but Wilson did not. However, they **both thought it was a moral duty** for the company to do something to help parents better manage work-life balance.
C(a)M(a) Causal codes aligned, moral codes aligned	In thinking independently and privately about this proposal, Price and Powell **agreed** that the project would **yield substantial carbon emission reductions**, bringing total emissions **far below the legally required threshold**. They also **both thought it was a moral duty** for the company to do as much as it can for the environment, including surpassing the legal emissions threshold.	In thinking independently and privately about this proposal, Smith and Wilson **agreed that an on-site daycare facility would serve to provide relief to parents**. Moreover, they **both thought it was a moral duty** for the company to do something to help parents better manage work-life balance.
C(m)M(m) Causal codes misaligned, moral codes misaligned	In thinking independently and privately about this proposal, Price and Powell **disagreed on whether the project would yield substantial carbon emission reductions**, bringing total emissions **far below the legally required threshold**. Price thought it would but Powell did not. They also had **different views about the moral implications** of the project. **Price thought it was a moral duty** for the company to do as much as it can for the environment, including reducing emissions below what is required by law but **Powell did not**.	In thinking independently and privately about this proposal, Smith and Wilson **disagreed on whether an on-site daycare facility would serve to provide relief to parents**. Smith thought it would but Wilson did not. They also had **different views about the moral implications** of the project. **Smith thought it was a moral duty** for the company to do something to help parents better manage work-life balance but **Wilson did not**.
No information condition	In thinking independently and privately about this proposal, Price and Powell **had differing opinions**.	In thinking independently and privately about this proposal, Smith and Wilson **had differing views**.

After reading each vignette, participants responded to an open-ended question inquiring about the root cause of the disagreement between the two individuals, the dependent variable items, a series of control measures, our instrument for attributing sources of disagreements to causal or moral codes from Study 1, and the intrateam conflict measure (Jehn and Mannix, 2001). The questionnaire concluded with questions about demographics, participants' opinions about climate change, and their current experience regarding daycare services offered by their employers.

### 4.3. Measures

We report Cronbach's alpha values for each measure in Table 2, separately for each vignette.

**Table 2:** Reliability of measures used in Study 2.

**Measure**	**# of items**	**α_daycare_**	**α_greentech_**
Perceived likelihood of reaching agreement	3	0.882	0.892
Perceived relationship conflict	3	0.850	0.880
Perceived negative affect between the parties	3	0.902	0.908
Likelihood of future engagement	3	0.933	0.916
Likelihood of developing a positive evaluation	3	0.855	0.872
Perceived moral code misalignment	4	0.918	0.946
Perceived causal code misalignment	4	0.933	0.932

**Dependent variables:** Participants viewed outcome measures in two separate blocks, both of which also included filler items. Different scale anchors were used in each block to facilitate participants' evaluation of the items. To test our hypotheses, we calculate mean scores by vignette for each dependent variable.^4^

**Relationship conflict** was measured with three items from the intra-team conflict measure used in Study 1 that we sourced from Jehn and Mannix (2001). Participants indicated their agreement with each item using a 5-point response scale (1-strongly disagree, 5-strongly agree), with higher values indicating greater conflict. A sample item is “They are experiencing tension in their relationship.”

**Likelihood of reaching an agreement** was measured with three items including “Reach a joint position on this matter,” “Come to an agreement on the proposal,” and “Resolve the differences in their opinions.” Participants indicated how likely they viewed each item to be using a 5-point response scale (1-Extremely unlikely, 5-Extremely likely). We recoded the responses during our analysis such that a higher score indicates less likelihood of reaching an agreement.

**Likelihood of negative affect developing between the parties** was measured with three items, which we developed based on other-condemning emotions previously identified by moral psychologists (Haidt, 2003; Brandt et al., 2019). Participants assessed whether the parties in the vignette were likely to feel disgust, contempt, and angry toward each other.

**Likelihood of avoiding future engagement**^5^ was measured with three items including “Be willing to collaborate in future projects,” “Want to work together again after this project,” and “Seek each other's opinion in the future.” We recoded the items such that higher values indicate a higher perceived likelihood of avoiding future engagement.

**Manipulation check and other measures:** We included our code misalignment scale from Study 1 to confirm that the manipulations functioned as expected (see Appendix 4 for details on factor analyses of the scale). In addition, we included three items along with the likelihood of negative affect development, which we intended to measure a more generalized evaluation between the parties. A sample item was “Have a generally favorable view of each other.” Finally, we included some exploratory items, including several adapted from the Behavioral Trust Inventory (Gillespie, 2003) and the team psychological safety measure developed by Edmondson (1999). These are not included in our theoretical framework and not reported in our analyses.

### 4.4. Analyses and results

#### 4.4.1. Manipulation checks

**Figure 3:**
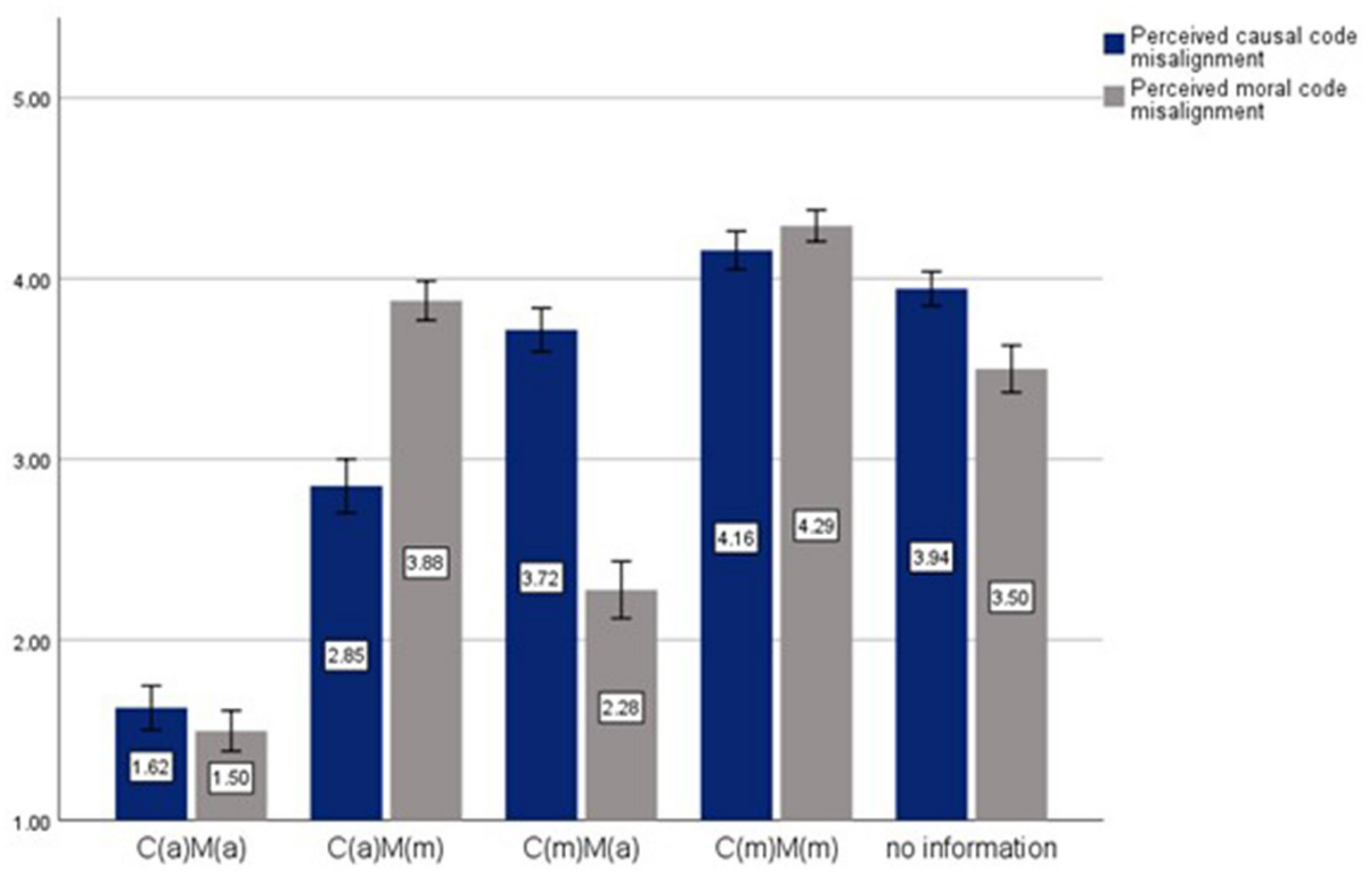
Study 2 manipulation check: perceived code misalignment by condition.

Figure 3 presents mean code misalignment attributions for each experimental condition. To confirm that our causal and moral code misalignment manipulations performed as expected, we conducted a set of *t*-tests for each vignette where we compared the aggregate mean code misalignment perceptions across conditions where the source of misalignment differed.

Specifically, in the daycare scenario, mean attributions to misalignments in moral codes in the moral code misalignment conditions [C(a)M(m) and C(m)M(m)] were significantly higher than those in the remaining three groups (*M* = 4.05, *SD* = 0.72 vs. *M* = 2.50, *SD* = 1.20), *t*_(473)_ = −16.62, *p* < 0.001. Participants were also significantly more likely to make attributions to moral code differences in these two conditions where they were informed of a moral code misalignment relative to the “no information condition” (*M* = 3.43, *SD* = 0.94), *t*_(306)_ = −6.344, *p* < 0.001). Moreover, participants in the causal code misalignment conditions [C(m)M(a) and C(m)M(m)] perceived significantly higher causal code misalignment compared to the remaining three groups [(*M* = 3.95, *SD* = 0.71) vs. (*M* = 2.86, *SD* = 1. 27), *t*_(473)_ = −10.81, *p* < 0.001]). However, we found no statistically significant differences in perceived causal code misalignment between the causal-code misalignment groups and the “no information” group (*M* = 4.06, *SD* = 0.62), *t*_(283)_ = 1.24, *p* = 0.215).

Similarly, in the case of the green technology vignette, attributions of disagreements to causal and moral code differences in the two groups where these codes were misaligned [C(m)M(a) and C(m)M(m) in the case of causal code differences and C(a)M(m) and C(m)M(m) in the case of moral code differences] were significantly higher than the remaining three groups' aggregate means [Causal code differences: *M* = 3.95, *SD* = 0.91 vs. *M* = 2.79, *SD* = 1.29, *t*_(473)_ = −10.774, *p* < 0.001; Moral code differences: *M* = 4.13, SD = 0.74 vs. *M* = 2.39, SD = 1.28, *t*_(473)_ = −16.843, *p* < 0.001]. As with the daycare vignette, comparing against the “no information” group yielded significant results for moral code differences (*M* = 3.57, *SD* = 0.88), *t*_(279)_ = −5.637, *p* < 0.001, but not for causal code differences (*M* = 3.83, *SD* = 0.69), *t*_(284)_= −1.147, *p* = 0.253.

As can be seen in Figure 3, the lack of a difference between perceptions of causal code misalignment in the “no information” control condition and the two treatment conditions with misaligned causal codes is partly due to participants perceiving a high degree of causal misalignment in the “no information” condition and partly due to them perceiving a lower degree of causal misalignment when the moral codes are aligned (these patterns are similar across the vignettes). The former may suggest that in the absence of specific information regarding the source of conflict, individuals tend to default to causal codes to “explain” the perceived conflict, an effect that we explore in Section 4.4.3 below. The latter is one instance of several spillover effects we find between perceptions of causal and moral code alignment.

In addition, we find the following spillover effects: In the daycare vignette, we find significantly higher causal attributions when comparing C(a)M(m) to C(a)M(a) [*M* = 2.70, *SD* = 0.98 vs. *M* = 1.71, *SD* = 0.93, *t*_(188)_ = −7.07, *p* < 0.001] and significantly higher moral attributions when comparing C(m)M(a) to C(a)M(a) [*M* = 2.39, *SD* = 0.10 vs. *M* = 1.55, *SD* = 0.10, *t*_(165)_ = −6.04, *p* < 0.001]. Similarly in the green technology vignette, we find significantly higher causal attributions when comparing C(a)M(m) to C(a)M(a) [*M* = 3.03, *SD* = 0.12 vs. *M* = 1.55, *SD* = 0.08; *t*_(187)_ = −10.78, *p* < 0.001] and significantly higher moral attributions when comparing C(m)M(a) to C(a)M(a) [*M* = 2.18, *SD* = 0.12 vs. *M* = 1.45, *SD* = 0.07; *t*_(192)_ = −5.36, *p* < 0.001]. As a whole, the results echo those of Study 1, showing that even though participants were reliably able to distinguish between causal and moral code misalignments, the presence of either misalignment led them to see more of the other.

#### 4.4.2. Hypothesis tests

To test our first hypothesis that misalignments in either causal or moral codes increase perceptions about how challenging a conflict will be to resolve, we conducted a series of regressions on the likelihood of conflict resolution, the likelihood of future engagement, perceived relationship conflict, and negative affect between the parties (Table 3). We use mixed (multi-level linear regression) models, performed on Stata 17 (StataCorp, 2021). These models pool data from both vignettes, estimate a participant-specific intercept, and report the variance as a random effect. We exclude the “no information” condition and control for vignette type in all models.

**Table 3:** Study 2 hypothesis tests: estimates from mixed effect regressions (*N* = 759).

	**Likelihood of conflict resolution**		**Likelihood of avoiding future engagement**		**Relationship conflict**		**Negative affect**	
**Fixed effects parameters**	β	***p***	β	***p***	β	***p***	β	***p***
Causal code misalignment only [C(m)M(a)]	0.582	< 0.001	0.778	< 0.001	1.115	< 0.001	0.618	< 0.001
Moral code misalignment only [C(a)M(m)]	0.874	< 0.001	1.133	< 0.001	1.416	< 0.001	0.907	< 0.001
Misalignment in both C and M [C(m)M(m)]	1.680	< 0.001	1.811	< 0.001	1.97	< 0.001	1.454	< 0.001
Vignette: green tech	0.072	0.18	0.054	0.317	−0.095	0.064	0.019	0.72
Intercept	1.483	< 0.001	1.576	< 0.001	1.64	< 0.001	1.514	< 0.001
**Random effects parameters**	**Estimate**	**SE**	**Estimate**	**SE**	**Estimate**	**SE**	**Estimate**	**SE**
σ^2^ (ID)	0.166	0.039	0.267	0.048	0.295	0.047	0.273	0.048
σ^2^ (res)	0.510	0.041	0.519	0.043	0.452	0.038	0.51	0.042
LR (1)	18.49	< 0.001	33.21	< 0.001	43.58	< 0.001	34.63	< 0.001
Wald *X*^2^ (4)	439.60	< 0.001	460.27	< 0.001	617.72	< 0.001	299.9	< 0.001
LL	−919.467		−966.966		−941.07		−964.881	
**Tests of H2 and H3:**								
*X*^2^(1) B_*C*(*m*)*M*(*a*)_ = B_*C*(*a*)*M*(*m*)_	12.2	< 0.001	16.24	< 0.001	12.74	< 0.001	10.87	0.001
*X*^2^(1) B_*C*(*m*)*M*(*m*)_ = B_*C*(*m*)*M*(*a*)_	175.22	< 0.001	139.79	< 0.001	104.37	< 0.001	92.29	< 0.001

We test H1 through the estimated effects of dummy variables for conditions with only causal code misalignment [C(m)M(a)] or moral code misalignment [C(a)M(m)] against the omitted category of no misalignment [C(a)M(a)]. Both variables have the expected effects on all dependent variables, supporting H1.

To test H2, we compare the coefficient estimates for the dummy variables corresponding to the C(m)M(a) and C(a)M(m) conditions (conditions where only one code is misaligned). As predicted, we find that moral code misalignments had a higher impact on the outcomes than causal code misalignments. These differences are significant for all DVs.

To test H3, we test the difference between the estimated effect for the C(m)M(m) (both codes in misalignment) condition and the C(m)M(a) (only causal codes misaligned) condition. Tests (presented in the last row) show that misalignment in both codes do have greater effects than misalignment only in causal codes, supporting H3.

In a supplemental analysis that we had not registered, we perform two sets of OLS models, one for each vignette (Appendix 5). While this reduces the sample size to half of what we had expected to provide adequate power in study design, it permits us to examine vignette-specific effects. Analyses support H1 and H3 for both vignettes. Differences in estimated effects of causal and moral code misalignments fail to reach conventional levels of statistical significance for the daycare vignette for the likelihood of conflict resolution, perceived relationship conflict, and negative affect between the parties. However, results remain directionally consistent.

Even though we had not hypothesized or registered it, we also test if misalignment in both types of codes increases the impact of having misalignment only in moral codes. Tests comparing the estimated effect for the C(m)M(m) (both codes in misalignment) condition and the C(a)M(m) (only moral codes misaligned) condition show that misalignment in both codes has a significantly greater effect than misalignment in moral codes alone for all dependent variables and this effect is observed in the multi-level analyses as well as OLS regressions for each vignette type.

In additional analyses with control variables (available upon request), we examine the effects of participant perceptions of importance of the issue to the managers featured in the vignette, strength of the managers' beliefs and opinions about the issue, how interdependent the managers' outcomes are, and how confident the managers are that their own beliefs are correct and the other has wrong beliefs. These variables are informed by the prior literature that finds outcome importance, actor interdependence, and evidentiary skew (parties' belief that the weight of evidence overwhelmingly supports their respective points of view) to be the principal antecedents of attitude polarization (Minson and Dorison, 2022). While these variables have statistically significant effects in some models, including them does not have appreciable effects on the results we have reported above. This indicates that perceptions of cultural misalignment are distinct from attitude polarization and strength (Howe and Krosnick, 2017).

We also examine the effect of perceptions of how open and receptive managers in the vignette perceive the other manager to be toward their ideas. This variable is highly correlated with our four dependent variables (*r* = −0.63 to −0.73) and is moderately correlated with the “both codes clash” condition (*r* = −0.36). Including it in the regression models makes the effect of causal misalignment statistically indifferent from zero, as well as statistically indifferent from the effect of moral misalignment. This suggests that perceptions of cultural misalignment and the effect they have on perceived likelihood of conflict resolution overlap at least partially with some processes documented in the moral conviction literature (Skitka et al., 2021).

Finally, we do not see incentive-compatibility as a potential problem for our studies, for a few reasons. First, we do not ask participants to provide their own opinions on a potentially conflictual topic (which might have created a problem in eliciting truthful responses). Second, we present the protagonists of our vignettes as employees of the same organization solving a business problem, so that participants would assume aligned incentives. Third, if the incentive we provided for participation in the study was not sufficient to elicit effort, we would see noise. That is, there is no reason to expect systematically different effects across conditions. Finally, even though there is no reason for participants to implicate themselves in the scenarios where they assume the role of observers, we did collect measures of potential personal investment in the questions of daycare provision (whether they have school age children and whether their employer provides daycare) and climate change (whether they believe climate change to be an urgent problem and whether they believe enough is being done on this matter). In regression models, we did not find these to affect our findings.

#### 4.4.3. Exploratory analysis

In pre-registered exploratory analysis of whether participants' attributions to causal or moral misalignments differ in the absence of any information about codes, we examine the manipulation checks in the “no information” group. Table 4 provides descriptive statistics. We find that when specific information regarding the source of conflict was not provided, participants made higher attributions to causal code misalignments (*M* = 3.94, *SD* = 0.66) than to moral code misalignments [*M* = 3.50, *SD* = 0.91, *t*_(190)_ = 6.686, *p* < 0.001], and this pattern held for each vignette. We find the same if we only focus on the first vignette that the participants saw, with perceived misalignment in causal codes (*M* = 3.90, *SD* = 0.66) greater than perceived misalignment in moral codes [*M* = 3.43, *SD* = 0.88, *t*_(107)_ = −5.918, *p* < 0.001]. Additionally, while perceived moral code misalignment does not vary between vignettes [*M*_*daycare*_ = 3.43, *SD*_*daycare*_ = 0.94; *M*_*greentech*_ = 3.57, *SD*_*greentech*_ = 0.88, *t*_(189)_ = 1.070, *p* = 0.286], causal code misalignment was higher for the daycare vignette (*M* = 4.06, *SD* = 0.62) compared to the green technology vignette [*M* = 3.83, *SD* = 0.69, *t*_(189)_ = 2.453, *p* = 0.015].

**Table 4:** Study 2 exploratory analysis of the “no information” condition.

	**Daycare (*****N*** = **95)**		**Green technology (*****N*** = **96)**		**Total**	
	***M***	**SD**	***M***	**SD**	***M***	**SD**
Perceived misalignment in moral codes	3.43	0.94	3.57	0.88	3.50	0.91
Perceived misalignment in causal codes	4.06	0.62	3.83	0.69	3.94	0.66
*t*-tests of differences in means	*t*_(94)_ = 6.99		*t*_(95)_ = 2.75		*t*_(190)_ = 6.69	

These results could be driven by the nature of the codes or their measurement. In the absence of specific guidance in the vignettes, participants may have emphasized causal code misalignments because items in the causal code misalignment subscale may have been perceived as more practical, proximate, or relevant to an organizational setting than moral code misalignment items. This should not be a concern within the treatment condition where both codes are in alignment. However, in that condition, the results reveal a similar pattern: participants perceive significantly higher causal code misalignment between the parties (*M* = 1.70, *SD* =0.88) than moral code misalignment (*M* = 1.57, *SD* = 0.81), *t*_(90)_ = −2.507, *p* = 0.014. That is, even participants who were told that the managers agreed on both causal and moral codes perceived some misalignment, and the misalignment they perceived in causal codes was greater than the misalignment they perceived in moral codes.

It is also interesting to consider what the participants in our studies thought about how to resolve the cultural conflicts we described for them. Using an analysis of text based on word embedding methods, we identified key themes in the open-ended responses from our participants to a question we asked in Study 2a about their proposed resolution mechanisms for each vignette. The results indicate that “mediation” is suggested as a mechanism for resolution in all cases except for pure moral code misalignment, and “research” or “statistical data” come up only in the case of pure causal code misalignment. This reiterates our findings from Study 1 that people find the distinction between misalignments in moral and causal codes to be meaningful and suggests that they also have theories about specific interventions that might work for each type of misalignment.

## 5. Discussion and conclusion

As pioneers of the Carnegie perspective recognized, conflicts in organizations are not limited to divergent interests rooted in misaligned incentives. As subsequent behavioral studies have shown, differences in representations alone (even when incentives are aligned) can create disagreements and conflict: “*Variations in perceptions may fuel debate concerning the best course of action in response to feedback* (Kaplan, 2008) *and may provide managers the chance to ‘self-enhance* (Jordan and Audia, 2012) *through over-favorable interpretation of feedback* (Joseph and Gaba, 2015). *Divergent interpretations may lead to disagreements about the best course of action or the evaluation of alternatives. For example, it might shape whether new opportunities are viewed as threats or opportunities* (Gilbert, 2005). *It may also lead to inaction as organizational members continually undo or reverse decisions already made* (Denis et al., 2011)” (Joseph and Gaba, 2020, p. 289).

We have built on this prior work to examine cultural conflicts as a distinct category of conflicts that can arise even when incentives are aligned. A hallmark of cultural conflicts is the difference in interpretation and evaluation of the same information across individuals and groups, which are in turn driven by differences in the pre-existing cognitive constructs across them. The key premise of this study is that resolution of such cultural conflicts should begin with a diagnosis of the sources of conflicts in cultural cognitions. This is likely to be useful for at least two reasons. First, different forms of cultural conflict may require different kinds of interventions to resolve, and diagnosis can help match the intervention to the problem. Second, some types of cultural conflict may just be easier to resolve, so that diagnosis can aid prioritization. To develop this line of reasoning, we propose that people (1) can perceive differences in the sources of cultural conflicts and (2) ascribe different levels of difficulty to resolving cultural conflicts arising from different sources.

We draw on the concept of cultural codes (Koçak and Puranam, 2023) to develop a simple basis for differentiating the sources of cultural conflict as perceived by observers (i.e., potential mediators)—into misalignments in moral and causal codes. Because moral codes allow for multiple dimensions of desirability, individuals may have additional objectives (and constraints), in addition to the rewards arising from incentives. Whether individuals share moral codes or not, they might also have differing beliefs about means–ends relationships (causal codes). Differences in moral *or* causal codes can produce cultural conflicts in organizations, and incentive alignment may not be sufficient for resolving cultural conflicts.

In Study 1, we find that study participants are receptive to this distinction between moral and causal codes and attribute sources of disagreement to each code accurately in line with our manipulations. In Study 2, we show that perceived misalignments in causal and moral codes both lead to heightened perceptions about how challenging a conflict will be. Furthermore, the joint presence of both kinds of misalignments amplifies the effect of each source on perceptions about how challenging a conflict will be to resolve. It is also the case that perceived misalignment of moral codes increases perceptions about how challenging a conflict will be to resolve to a greater extent than misalignment of causal codes. Put simply, if observers believe a cultural conflict arises from differences in moral codes, they may not even see it as worthwhile to attempt a resolution.

Our findings point to two classes of interventions that mediators can implement to resolve cultural conflicts. First, preventing misdiagnosis of conflicts as arising from misaligned moral codes and focusing public debates on causal code misalignments before issues become moralized can help overcome some disagreements that will otherwise appear intractable. In this way, we offer a connection to the literature on conflict and negotiation, which already offers rich insights into how cultural cognitions impact the inputs, processes, and outputs of negotiations within and across social groups (Gunia et al., 2016). We suggest that future research might attempt to identify optimal tactics for conflict resolution (such as moral suasion vs. appeals to scientific analyses), contingent on whether these arise from misalignments in causal or moral codes.

A second possibly more controversial intervention is to reframe conflicts that arise from either kind of misalignment as being primarily about causality (perhaps when codes are fuzzy and it is genuinely unclear as to what the underlying truth of the matter is). This focuses efforts toward resolution, which would not even be undertaken if the source of misalignment was perceived to be primarily differences in moral codes. It does not guarantee resolution, but rather an effort toward resolution.

A third intervention can be aimed not at resolving cultural conflict but rather at stimulating useful kinds of conflict. For instance, one may compose groups of individuals selected to be homogenous on moral codes but not on causal codes—so that the resulting diversity of views on the links between causes and consequences may promote innovation and creativity, whereas the converse may not.

These interventions are likely to be most relevant for collective decision-making, where multiple parties need to make a joint decision in a committee-like structure. Thus, our research helps advance prior recommendations to improve the effectiveness of strategy-formulation meetings by separating objectives and the roadmaps to achieve them (Bourgoin et al., 2018) or by using strategy mapping tools to debate strategic options (Carroll and S*ϕ*rensen, 2021). They are likely to be of greatest use in situations where agents individually or collectively hold multiple goals (Ethiraj and Levinthal, 2009; Gaba and Greve, 2019; Audia and Greve, 2021).

Going forward, a fruitful follow-up to our study would be to examine the effect of attributions made by agents that directly participate in a conflict. As prior literature shows, individuals experiencing a conflict make inferences about how likely they are to resolve their disagreement and this in turn shapes their behavior (Minson and Dorison, 2022). Our study suggests that these inferences will be shaped by whether individuals perceive misalignments in causal or moral codes to be at the core of their disagreements. However, our finding that third parties can make such diagnoses does not imply that active participants in a conflict can do the same. Third parties might more easily remove themselves from the “hot” emotions of a conflict situation and make more attributions to misalignments in causal rather than moral codes. That said, our findings that even third-party attributions carry some spillover effects (seeing moral code differences where we only say there are causal code differences and the reverse) suggest that the same might happen with parties to a conflict.

The spillover effects we find may more generally explain why causal and moral misalignments might remain tangled in ordinary life. They might point to a type of “halo effect” (previously shown for judgments of individual character, e.g., Judd et al., 2005) that pertains to relationships, whereby a pair's failure to agree in one (causal or moral) domain creates a perception of misalignment in the other domain. They might also stem from lay theories about cultural codes. We are unable to examine the reasons for spillover effects in this study the way the halo effect has been examined (Stellar and Willer, 2018) and we leave it to future work.

Another promising direction for future studies is to examine how the moralization of issues in public discourse might impact attributions and the effect of attributions on perceptions of conflict resolution. The stronger tendency we found in our studies to infer causal code misalignments than moral code misalignments is not universal. For instance, in recent years, we have seen some disagreements that appear to be resolvable through scientific research to instead become fodder for “culture wars” (Macy et al., 2019; Broćić and Miles, 2021). The COVID-19 pandemic saw debates about mask mandates in some countries stay centered on the efficacy of masks for preventing contagion, whereas in others they evolved to pit value for personal freedom (defended by one party) against value of public concern (defended by another party). Debates on how to address climate change have undergone a similar transformation in some settings, from a technological problem to a moralized and politicized issue. We can expect H2 and H3 to be even more strongly supported for issues that are moralized or politicized.

In sum, the systematic study of cultural conflicts within organizations is at a nascent stage. The theme is relevant particularly to organizations attempting to balance disparate objectives such as social impact and profitability, but also more generally to any organization that is not monocultural. We believe our approach to modeling differences in cultural codes in terms of morality and causality can be useful to develop this agenda further.

## Data availability statement

The original contributions presented in the study are included in the article/Supplementary material, further inquiries can be directed to the corresponding author.

## Ethics statement

The studies involving humans were approved by INSEAD Institutional Review Board. The studies were conducted in accordance with the local legislation and institutional requirements. The participants provided their written informed consent to participate in this study.

## Author contributions

ÖK, PP, and AY contributed to conception and design of the study, performed statistical analyses, and wrote sections of the manuscript. All authors contributed to manuscript revision, read, and approved the submitted version.

## Footnotes

^1^In this usage, we differ from research on moral psychology, where the term “moral” has a socially prescriptive component and typically refers to other-regarding values that are perceived to set universal standards of conduct (Skitka et al., 2021). Our usage of the term is broader and includes what this literature refers to as “social conventions.” Also note that we do not assume different dimensions of desirability (e.g., financial performance and social impact) to be commensurable. In other words, moral codes may not be reducible to utility functions. This is why different interests may not be resolved through incentive design.

^2^This distinction we make between causal and moral codes is similar to the distinction Haidt and Kesebir (2008) make between plain facts and anthropocentric facts, the latter being facts whose truth value depends on moral, ontological, religious, linguistic, legal, metaphysical, or political reasoning about the social world and thus can only be defended with reference to a cultural system. The distinction also roughly reflects the difference between “beliefs” and “values”—roughly, because (a) “beliefs” and “values” have been used in reference to slightly different concepts across different research programs and (b) codes are mappings, whereas beliefs and values need not be (see Koçak and Puranam, 2023).

^3^In contrast with “logic of consequences,” “logic of appropriateness” (or “rule based action”) corresponds to use of codes based on associated situations or identities, rather than likelihood and value of potential consequences (March and Olsen, 1989; March and Simon, 1993; March and Olsen, 2011). For instance, a parents' representative might advocate for any measure that is intended to support parents, regardless of the likelihood of its success or other effects. The logic of appropriateness is a form of deontological reasoning, and while it can be represented through particular codes, we defer a deeper discussion of this to future work.

^4^Exploratory factor analyses of the pooled items for each vignette show a factor structure in line with our expectations for the green technology vignette. However, items relating to relationship conflict and negative affect did not distinguish from each other for the daycare vignette.

^5^An additional item “Want to avoid each other in the future” was included as part of this construct. However, in exploratory factor analyses, this item exhibited high factor loadings with both this factor as well as likelihood of negative affect development. Thus, we omitted it in our calculations of dependent measures.
