# Supplementary material for: Decoding cultural conflicts
Source: Front Psychol. 2023 Sep 14;14:1166023. doi: 10.3389/fpsyg.2023.1166023 (PMC10538637; doi:10.3389/fpsyg.2023.1166023)
Supplement: Supplementary file 1 [file Data_Sheet_1.docx]

**Appendix 1 – Scale Validation in Study 1 (Distal Scale)**

The instrument to measure attributions to code misalignments was developed by the authors and aimed to capture attributions of disagreements to misalignments in causal versus moral codes. We constructed two versions of the instrument, at two levels: distal/abstract and proximate/concrete. Though our goal was to ultimately develop an instrument that could be used across a range of topical areas, we also developed the proximate version to test whether presenting the source of disagreements in distal/abstract versus proximate/concrete terms makes a difference in attributions and to use this domain-specific instrument to assess the convergent validity of our distal measure.

The items were generated through iterative discussions among the authors and revised to ensure that they were clear, concise, and not ‘double-barreled’ (Hinkin, 1998). Though there is no specific guideline with respect to the appropriate number of items per construct, three items have been shown to be sufficient for obtaining internal consistency reliabilities (Hinkin, 1998). To ensure that we would have a sufficient number retained, we generated four items each for causal and moral code misalignment attributions.

All analyses of the code misalignment instrument were conducted on Jamovi 2.2 (The Jamovi Project, 2021). We performed exploratory factor analyses, followed by confirmatory factor analyses. We first report analyses on the distal scale, followed by analyses on the proximate scale. Given our results, as well as our primary goal of developing an instrument that could be used across a range of topical areas, we focused our additional investigations on the distal measure and used it in Study 2.

**Distal Scale Analyses**

Since the instrument was newly developed, our first step was to assess the item structure with exploratory factor analysis. The Kaiser-Meyer-Olkin index (*KMO* = 0.798) was acceptable and Bartlett’s test of sphericity was significant, *χ^2^*(28) = 338, *p* < .001, allowing us to proceed with an EFA. We used principal axis factoring with direct oblimin rotation and relied on parallel analysis (Horn, 1965; Dinno, 2009) to determine the number of factors extracted. None of the items exhibited cross-loading and all factor loadings were above 0.400. Thus, we retained all items in the final solution, which yielded two factors representing moral and causal misalignment (Table A1-1).

We additionally conducted a confirmatory factor analysis of our intended 2-factor structure to assess goodness of fit. The model yielded good fit, CFI = 0.998, RMSEA = 0.017, *χ^2^*(19) = 19.50, *p* = 0.425. by common acceptance levels (Ullman, 2006; Hu and Bentler, 1998, 1999; Bentler, 1990). Standardized factor loadings ranged between 0.593 and 0.895, with only two estimates below the conventional standard of 0.700. Average variance extracted was 63.5% for moral code misalignment and 55.2% for causal code misalignment. Scale reliabilities, reported in the main text, were above 0.700. Finally, the sub-scales exhibited good reliability (*α_causal_* = 0.820, *α_moral_* = 0.872). Taken together with the fit indices, these results suggest adequate evidence of convergent validity (Hair et al., 2010).

To assess whether our items were substantially differentiated from intrateam conflict dimensions in extant literature, we repeated the exploratory factor analysis while including Jehn and Mannix’s (2001) intrateam conflict scale, which consists of three items each for task conflict, process conflict, and relationship conflict. Several items from this latter scale exhibited low loadings (below 0.400) and one item cross-loaded onto more than one factor. We performed iterative extractions, dropping poorly performing items until the solution in Table A1-2 was obtained. The remaining items in the intrateam conflict scale included all three relationship conflict items and one task conflict item. The final factor structure has three dimensions - moral code misalignment, causal code misalignment, and intrateam conflict - lending support to our suggestion that code conflicts are distinct from types of intrateam conflict studied in prior literature.

Though the results of this exploratory factor analysis support the claim that our code conflict measure is distinct from the extant measures of intrateam conflict, we undertook several additional analyses to better assess convergent and discriminant validity. First, we investigated correlation patterns (Table A1-3) among the code misalignment and intrateam conflict dimensions. Causal code misalignment is not significantly correlated with either process-based or relationship-based team conflict measures and it has a small positive correlation with task-based team conflict (*r* = 0.203, *p* = 0.048). This suggests that causal code misalignment is distinct from previously validated measures of team conflict. Though moral code misalignment was positively correlated with all facets of intrateam conflict, correlation coefficients do not exceed 0.460.

Second, we conducted additional confirmatory factor analyses using an independent sample (n=229) to further validate the moral and causal code misalignment scales. The data was collected through Prolific as part of a larger research program. The preregistration for this data collection effort is available on OSF (<https://osf.io/enjz2/?view_only=6113bede66674d3fb96c767d97283ce6>). The survey employed the same daycare vignette as Study 1 with some adjustments to explore potential moderators and collected both distal and proximate measures. Confirmatory factor analyses with the distal misalignment scale show that average variance extracted was slightly below the recommended threshold of 0.50 (moral code misalignment: 0.48, causal code misalignment: 0.44). However, fit indices suggest that the overall fit of the model was good (CFI = 0.978, TLI = 0.967, RMSEA = 0.049). Additionally, we conducted a series of confirmatory factor analyses where we included the intrateam conflict dimensions and evaluated model fit. Table A1-4 summarizes model fit statistics. A 5-factor model distinguishing code misalignments and dimensions of intrateam conflict shows the best overall fit. Correlations between factors are reported in Table A1-5. Overall, these results confirm that our 2-factor instrument has acceptable convergent and discriminant validity.

| **Table A1-1. Instrument to measure attributions of disagreements to differences in causal and moral codes (Distal) (Study 1).** | | |
| --- | --- | --- |
| **Items** | **Factor 1** | **Factor 2** |
| They disagree because they differ in their moral convictions. | **0.85** | 0.01 |
| They disagree because they have conflicting values. | **0.79** | -0.02 |
| They disagree because they are incompatible in terms of their principles. | **0.78** | 0.11 |
| They disagree because their views on which course of action is morally appropriate are in contradiction. | **0.77** | -0.1 |
| They disagree about the consequences of their respective proposed actions. | -0.03 | **0.88** |
| They disagree because their views on the results of different courses of action are in contradiction. | 0.06 | **0.81** |
| They disagree about the causes and consequences of their proposed actions. | 0.04 | **0.68** |
| They disagree on what will happen if their respective proposed actions are implemented. | -0.18 | **0.55** |
| Note. N = 95. 'Principal axis factoring' extraction method was used in combination with an ‘oblimin’ rotation. Factor loadings above 0.300 are in bold. | | |

| **Table A1-2. Intrateam conflict scale and instrument to measure attributions of disagreements to misalignments in causal and moral codes (Distal) (Study 1).** | | | | | | | | |
| --- | --- | --- | --- | --- | --- | --- | --- | --- |
| **ITEMS** | **Factor 1** | **Factor 2** | **Factor 3** |  |  | |  |  |
| They disagree because they have conflicting values. | **0.89** | -0.01 | -0.13 |  |  | |  |  |
| They disagree because they are incompatible in terms of their principles. | **0.76** | 0.09 | 0.03 |  |  | |  |  |
| They disagree because they differ in their moral convictions. | **0.74** | -0.02 | 0.18 |  |  | |  |  |
| They disagree because their views on which course of action is morally appropriate are in contradiction. | **0.68** | -0.13 | 0.15 |  |  | |  |  |
| They disagree about the consequences of their respective proposed actions. | -0.02 | **0.88** | -0.03 |  |  | |  |  |
| They disagree because their views on the results of different courses of action are in contradiction. | 0.01 | **0.82** | 0.08 |  |  | |  |  |
| They disagree about the causes and consequences of their proposed actions. | 0.06 | **0.67** | -0.05 |  |  | |  |  |
| They disagree on what will happen if their respective proposed actions are implemented. | -0.18 | **0.54** | -0.01 |  |  | |  |  |
| They are experiencing emotional conflict. | 0.04 | -0.04 | **0.77** |  |  | |  |  |
| They are experiencing tension in their relationship. | 0.10 | 0.09 | **0.60** |  |  | |  |  |
| They get angry with each other. | -0.10 | -0.13 | **0.57** |  |  | |  |  |
| They have conflicting ideas about their work. | 0.08 | 0.21 | **0.51** |  |  | |  |  |
| Note. N = 95. 'Principal axis factoring' extraction method was used in combination with an ‘oblimin’ rotation. Factor loadings above 0.300 are in bold. | | | | | |  | |  |

| **Table A1-3. Correlations of Team Conflict and Code Misalignment Measures (Study 1)** | | | | | | | |  |
| --- | --- | --- | --- | --- | --- | --- | --- | --- |
| **Scale** | M (SD) | I | II | III | IV | V | VI | VII |
| I. Distal moral code misalignment (DMCM) | 2.96 (1.08) | 1 |  |  |  |  |  |  |
| II. Distal causal code misalignment (DCCM) | 3.60 (0.94) | -0.203* | 1 |  |  |  |  |  |
| III. Proximate moral code misalignment (PMCM) | 2.94 (1.11) | 0.815* | -0.301* | 1 |  |  |  |  |
| IV. Proximate causal code misalignment (PCCM) | 3.50 (0.90) | -0.387* | 0.676* | -0.387* | 1 |  |  |  |
| V. Intra-team task conflict (TC) | 3.08 (0.80) | 0.413* | 0.203* | 0.331* | 0.090 | 1 |  |  |
| VI. Intra-team relationship conflict (RC) | 2.46 (0.82) | 0.469* | -0.067 | 0.407* | -0.110 | -0.490* | 1 |  |
| VII. Intra-team process conflict (PC) | 3.00 (0.87) | 0.460* | 0.065 | 0.427* | -0.054 | 0.482* | 0.448* | 1 |
| VIII. Likelihood of conflict resolution | 3.22 (0.98) | -0.124 | -0.021 | -0.086 | -0.114 | -0.085 | -0.203* | -0.004 |
| * p < .05 |  |  |  |  |  |  |  |  |

| **Table A1-4. Results of Confirmatory Factor Analysis (Independent Sample)** | | | | | | |
| --- | --- | --- | --- | --- | --- | --- |
| **Model** | *χ2* | *df* | CFI | TLI | RMSEA | *Δχ2(df)* |
| 1-factor model: CCC&MCC&TC&RC&PC | 540.49 | 119 | 0.576 | 0.515 | 0.124 |  |
| 2-factor model: CCC&MCC, TC&RC&PC | 389.16 | 118 | 0.727 | 0.685 | 0.100 | 151.33 (1), p<0.001 |
| 3-factor model: CCC, MCC, TC&RC&PC | 200.482 | 116 | 0.915 | 0.900 | 0.056 | 188.68 (2), p<0.001 |
| 5-factor model: CCC, MCC, TC, RC, PC | 158.73 | 109 | 0.950 | 0.938 | 0.045 | 41.75 (7), p <0.001 |
| *Note.* CCC = causal code conflict scale; MCC = coral code conflict scale; PC = process conflict scale; RC = relationship conflict scale; TC = task conflict scale. | | | | | | |

| **Table A1-5. Code misalignment and team conflict dimensions correlation matrix (Independent Sample)** | | | | |
| --- | --- | --- | --- | --- |
| **Scale** | I | II | III | IV |
| I. Moral code misalignment | 1 |  |  |  |
| II. Causal code misalignment | 0.187 * | 1 |  |  |
| III. Task conflict | 0.283* | 0.187* | 1 |  |
| IV. Process conflict | 0.251* | 0.125 | 0.618* | 1 |
| V. Relationship conflict | 0.422 | 0.204* | 0.528* | 0.466* |
| * *p* < .05 |  |  |  |  |

**Appendix 2 – Analyses with the Proximate Scale in Study 1**

**Validation of the proximate scale**

The Kaiser-Meyer-Olkin index (*KMO* = 0.756) was acceptable and Bartlett’s test of sphericity was significant, *χ^2^*(28) = 229.95, *p* < .001. We used the same approach as above. Table 2 reports the factor loadings extracted. None of the items exhibited cross-loading and all factor loadings were above 0.400.

A confirmatory factor analysis of the code misalignment scales with our intended 2-factor structure yielded marginal model fit by common acceptance levels (Ullman, 2006; Hu and Bentler, 1998, Bentler, 1990), CFI = 0.928, RMSEA = 0.093, *χ^2^*(19) = 34.47, *p* = 0.016. Standardized factor loadings ranged between 0.390 and 0.826, though most estimates were below the conventional standard of 0.700, in particular for the causal scale. Average variance extracted was 53.1% for moral code misalignment and 37.2% for causal code misalignment. Scale reliability, indicated by Cronbach’s alpha, was good for the moral subscale (*α*_moral_ = 0.816) but only marginally acceptable for the causal subscale (*α*_causal_ = 0.684). Taken together with the fit indices, these results provide marginal evidence of convergent validity (Hair et al., 2010).

As with the distal scale, we further investigated the validity of the instrument using the independent sample discussed above. Though the fit indices were at acceptable levels (CFI = 0.949, TLI = 0.924, RMSEA = 0.064), the average variance extracted was well below the recommended threshold of 0.50 for both factors (moral code misalignment: 0.42, causal code misalignment: 0.37). These results suggest that the proximate version of our scale, though marginally valid, is a poorer instrument compared to the distal scale.

**Mean Attributions to Code Differences by Condition (Proximate) (Study 1)**

We compared mean attributions to causal and moral code differences across conditions in Study 1 using our proximate measure. These comparisons revealed similar patterns as the distal scale. Mean attributions by condition are reported in Figure A2. Participants in the causal code difference condition attributed the disagreements to misalignments in causal codes significantly more (*M* = 4.03, *SD* = 0.64) than participants in the moral code difference condition (*M* = 3.13, *SD* = 0.88), *t*(93) = 5.44, *p* <0 .001. In contrast, participants in the moral code difference condition showed a significantly higher tendency to attribute disagreements to misalignments in moral codes (*M* = 3.53, *SD* = 0.87) compared to participants in the causal code difference condition (*M* = 2.10, *SD* = 0.84), *t*(93) = 8.02, *p* < 0.001. Comparing scale scores within each condition, we found causal code misalignment scores to be significantly higher than moral code misalignment scores in the causal code difference condition, Δ = 1.93, t(38) = 10.49, p < 0.001. Similarly, there was a significant difference in the moral code difference condition such that moral code misalignment was perceived higher than causal code misalignment, Δ = 0.40, t(55) = 2.35, p = 0.023.

| \| **Table A2. Instrument to measure attributions of disagreements to misalignments in causal and moral codes (Proximate) (Study 1).** \| \| \| \| \| \| \| \| \| --- \| --- \| --- \| --- \| --- \| --- \| --- \| --- \| \| **ITEMS** \| **Factor 1** \| **Factor 2** \|  \|  \| \| \| They disagree about whether it is morally appropriate to open a day care center on company premises. \| **0.84** \| 0.05 \|  \|  \| \| \| They disagree about whether it is morally acceptable for a company to offer day care for its employees' kids. \| **0.77** \| 0.04 \|  \|  \| \| \| They disagree because they have incompatible views on whether operating a day care center is a righteous course of action. \| **0.66** \| -0.13 \|  \|  \| \| \| They disagree because they support different principles on what a company should do for its employees. \| **0.57** \| -0.08 \|  \|  \| \| \| They disagree because they expect different consequences to follow from a company owned day care center. \| 0.01 \| **0.75** \|  \|  \| \| \| They disagree because they hold divergent opinions about the outcome of operating a day care center on company premises. \| 0 \| **0.63** \|  \|  \| \| \| They disagree because they have different views about how operating a day care center will impact the company. \| -0.08 \| **0.58** \|  \|  \| \| \| They disagree because they have different opinions about the effectiveness of opening a day care center for employees’ children. \| 0.07 \| **0.43** \|  \|  \| \| \| Note. N = 95. 'Principal axis factoring' extraction method was used in combination with an ‘oblimin’ rotation. Factor loadings above 0.300 are in bold. \| \| \| \| \|  \| \|  \| |
| --- | --- | --- | --- | --- | --- | --- | --- | --- | --- | --- | --- | --- | --- | --- | --- | --- | --- | --- | --- | --- | --- | --- | --- | --- | --- | --- | --- | --- | --- | --- | --- | --- | --- | --- | --- | --- | --- | --- | --- | --- | --- | --- | --- | --- | --- | --- | --- | --- | --- | --- | --- | --- | --- | --- | --- | --- | --- | --- | --- | --- | --- | --- | --- | --- | --- | --- | --- | --- | --- | --- |

**Figure A2 – Study 1 Results: Perceived Code Misalignment by Condition (Proximate Scale)**


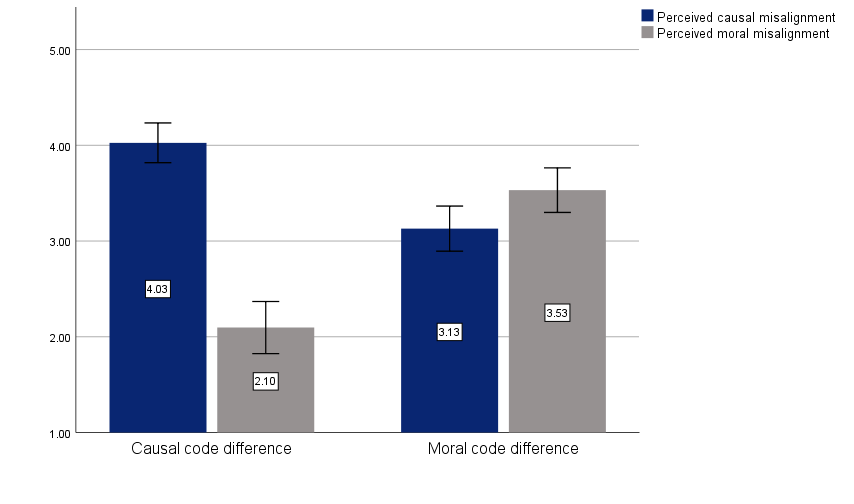


**Appendix 3 – Sample Details and Summary Results of Study 2a**

**Sample:** Participants were recruited through the Prolific platform. 496 participants completed the survey. We discarded 33 responses because they failed to correctly respond to at least one of three attention check questions, leaving a final sample size of 463 responses. The sample ranged between the ages of 18 and 80 (*M =* 37.02, *SD* = 12.67) and predominantly identified as white (n = 340). There were 226 male and 225 female participants, the remaining identified as non-binary (n = 9). Only 49 participants worked in an organization that offered childcare service or other type of support. Majority of participants (n = 295) considered climate change to be a global emergency and believed that the world should urgently do everything necessary to combat it. 48 participants did not consider climate change to be an emergency.

**Analyses and Results**: We measured our outcome variable with 3 items (perceived difficulty of reaching an agreement, extent of conflict experienced, and desirability of future collaboration). We also included the code misalignment scale to confirm that our manipulations performed as expected. Scale reliabilities for all three measures are reported in Table A3.1. All scales exhibited reliabilities above conventionally accepted levels.

We conducted exploratory and confirmatory factor analyses on the code misalignment scale for each vignette. Table A3.2.a and A3.2.b report the extracted structure from our exploratory factor analyses. In confirmatory factor analyses, a two-factor structure yielded acceptable fit for both vignettes (Daycare: CFI = 0.963, RMSEA = 0.107, χ^2^(19) = 119.05, *p* < 0.001; Greentech: CFI = 0.975, RMSEA = 0.094, χ^2^(19) = 96.26, *p* < 0.001).

Mean moral code misalignment and causal code misalignment attributions by condition are presented in Figure A3. (Note that Study 2a, unlike Study 2b, did not include a ‘no information’ control condition.) We conducted a set of t-tests to confirm that our causal and moral code misalignment manipulations performed as expected where we compared the aggregate mean code misalignment perceptions across conditions where the source of misalignment differed.

In the daycare scenario, mean attributions to misalignments in moral codes in the moral code misalignment conditions (C(a)M(m) and C(m)M(m)) were significantly higher than those in the remaining two groups (*M* = 3.91, *SD* = 0.69 versus *M* = 2.12, *SD* = 1.04), *t*(461) = -22.00, *p* < 0.001. Similarly, participants in the causal code misalignment conditions (C(m)M(a) and C(m)M(m)) perceived significantly higher causal code misalignment compared to the remaining two groups ((*M* = 3.82, *SD* = 1.09) versus (*M* = 2.28, *SD* = 1. 09), *t*(461) = -18.04, *p* < 0.001).

The results were similar for the green technology scenario where mean attributions to misalignments in moral codes in the moral code misalignment conditions C(a)M(m) and C(m)M(m)) were significantly higher than those in the remaining two groups (*M* = 3.99, *SD* = 0.73 versus *M* = 1.90, *SD* = 0.95), t(461) = -26.79, *p* < 0.001. Moreover, participants in the causal code misalignment conditions (C(m)M(a) and C(m)M(m)) perceived significantly higher causal code misalignment compared to the remaining two groups ((*M* = 4,01, *SD* = 0.72) versus (*M* = 2.32, *SD* = 1. 11), *t*(461) = -19.23, *p* < 0.001).

We constructed participant scores for our main dependent variable, “likelihood of conflict resolution” by averaging our three related items. Our measures were recoded such that higher values indicate lower likelihood of conflict resolution. For individual items of our outcome measure, higher values indicate greater perceived difficulty of reaching an agreement, higher conflict experienced, and lower desirability of future collaboration. We used mixed (multi-level linear regression) models, performed on Stata 17 (Stata Corp, 2021) to test our hypotheses. We controlled for vignette type in all models. Our results are summarized in Table A3.2. In addition to the results of our hypotheses tests using the dependent variable “likelihood of conflict resolution”, we report separate post hoc tests of each outcome measure item.

We tested H1 through the estimated effects of dummy variables for conditions with only causal code misalignment (C(m)M(a)), or moral code misalignment (C(a)M(m)) against the omitted category of no misalignment (C(a)M(a)). Both dummy variables have the expected effects on the dependent variable, supporting H1. The results were similar in our post hoc tests for each outcome measure item.

We compared the coefficient estimates for our dummy variables (C(m)M(a) and C(a)M(m) conditions) to test for H2 but failed to find support. However, we found misalignment in moral codes to have a marginally stronger effect on desire to collaborate in the future and misalignment in causal codes to have a stronger effect on perceived difficulty of reaching an agreement. There was no difference between conditions on experience of conflict.

Though not registered, we also conducted tests for H3 whereby we tested the difference between the estimated effect for the C(m)M(m) (both codes in misalignment) condition and the C(m)M(a) (only causal codes misaligned) condition. We found misalignment in the two codes together to have a greater effect on all three items relative to misalignment in causal codes alone.

**Figure A3 – Study 2a: Perceived Code Misalignment by Condition**


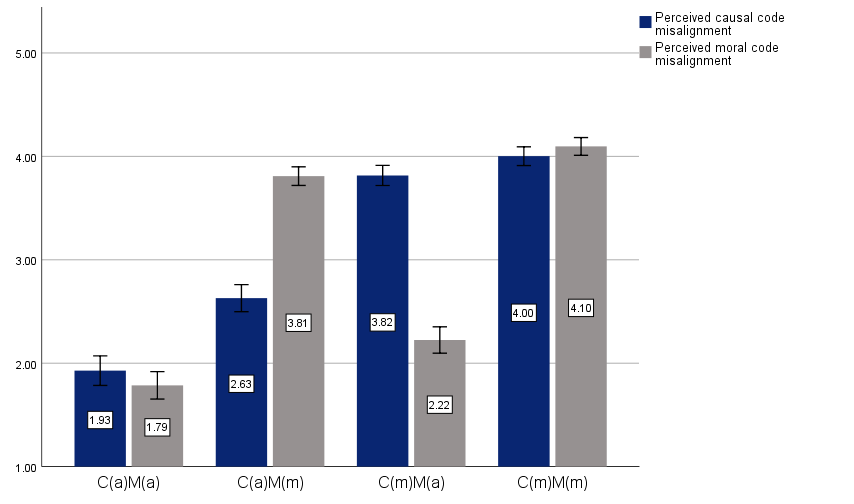


**Table A3.1. Reliability of measures used in Study 2A**

| Measure | # of Items | α_daycare_ | α_greentech_ |
| --- | --- | --- | --- |
| Likelihood of conflict resolution | 3 | 0.873 | 0.865 |
| Perceived moral code misalignment | 4 | 0.906 | 0.938 |
| Perceived causal code misalignment | 4 | 0.916 | 0.914 |

| **Table A3.2.a. Instrument to measure attributions of disagreements to misalignments in causal and moral codes (Daycare vignette) (Study 2a).** | | |
| --- | --- | --- |
| **ITEMS** | **Factor 1** | **Factor 2** |
| They disagree about the consequences of their respective proposed actions. | **0.89** | 0.00 |
| They disagree on what will happen if their respective proposed actions are implemented. | **0.88** | -0.07 |
| They disagree about the causes and consequences of their proposed actions. | **0.84** | 0.06 |
| Their views on the results of different courses of action are in contradiction. | **0.82** | 0.04 |
| They have conflicting values. | 0.07 | **0.85** |
| They are incompatible in terms of their principles. | 0.15 | **0.72** |
| They differ in their moral convictions. | -0.13 | **0.96** |
| Their views on which course of action is morally appropriate are in contradiction. | 0.06 | **0.79** |
| Note. N = 463. 'Principal axis factoring' extraction method was used in combination with an ‘oblimin’ rotation. Factor loadings above 0.300 are in bold. | | |

| **Table A3.2.b. Instrument to measure attributions of disagreements to misalignments in causal and moral codes (Greentech vignette) (Study 2a).** | | | |
| --- | --- | --- | --- |
| **ITEMS** | **Factor 1** | **Factor 2** |  |
| They disagree about the consequences of their respective proposed actions. | 0.01 | **0.88** |  |
| They disagree on what will happen if their respective proposed actions are implemented. | -0.12 | **0.91** |  |
| They disagree about the causes and consequences of their proposed actions. | 0.10 | **0.80** |  |
| Their views on the results of different courses of action are in contradiction. | 0.09 | **0.80** |  |
| They have conflicting values. | **0.90** | 0.03 |  |
| They are incompatible in terms of their principles. | **0.82** | 0.09 |  |
| They differ in their moral convictions. | **0.98** | -0.09 |  |
| Their views on which course of action is morally appropriate are in contradiction. | **0.84** | 0.05 |  |
| Note. N = 463. 'Principal axis factoring' extraction method was used in combination with an ‘oblimin’ rotation. Factor loadings above 0.300 are in bold. | | | |

| **Table A3.2. Study 2a Hypothesis Tests: Estimates from Mixed Effect Regressions (N=926)** | | | | | | | | |
| --- | --- | --- | --- | --- | --- | --- | --- | --- |
|  | **Likelihood of conflict resolution (average of 3 items)** | | **Perceived difficulty of reaching an agreement** | | **Desirability of future collaboration** | | **Extent of conflict experienced** | |
| **Fixed effects parameters** | **B** | **p** | **B** | **p** | **B** | **p** | **B** | **p** |
| Moral code misalignment only [C(m)M(a)] | 1.110 | <0.001 | 1.313 | <0.001 | 1.090 | <0.001 | 0.941 | <0.001 |
| Causal code misalignment only [C(a)M(m)] | 1.148 | <0.001 | 1.530 | <0.001 | 0.961 | <0.001 | 0.943 | <0.001 |
| Misalignment in both C and M [C(m)M(m)] | 1.973 | <0.001 | 2.447 | <0.001 | 1.783 | <0.001 | 1.684 | <0.001 |
| Vignette: green tech | 0.028 | 0.507 | 0.047 | 0.419 | -0.033 | 0.476 | 0.071 | 0.130 |
| Intercept | 4.414 | <0.001 | 1.549 | <0.001 | 1.610 | <0.001 | 1.601 | <0.001 |
|  |  |  |  |  |  |  |  |  |
| **Random Effects Parameters** | **Estimate** | **SE** | **Estimate** | **SE** | **Estimate** | **SE** | **Estimate** | **SE** |
| σ^2^(ID) | 0.063 | 0.023 | 0.044 | 0.039 | 0.225 | 0.036 | 0.110 | 0.029 |
| σ^2^ (res) | 0.423 | 0.028 | 0.774 | 0.051 | 0.503 | 0.033 | 0.504 | 0.033 |
|  |  |  |  |  |  |  |  |  |
| LR (1) | 7.59 | 0.003 | 1.30 | 0.127 | 45.94 | <0.001 | 14.83 | <0.001 |
| Wald *χ^2^* (4) | 933.47 | <0.001 | 847.94 | <0.001 | 557.93 | <0.001 | 544.97 | <0.001 |
| LL | -975.809 | | -1220.443 | | -1144.277 | | -1080.889 | |
|  |  |  |  |  |  |  |  |  |
| **Tests of H2 and H3:** |  |  |  |  |  |  |  |  |
| *χ^2^*(1) β_C(m)M(a)_=β_C(a)M(m)_ | 0.36 | 0.551 | 6.77 | 0.009 | 2.96 | 0.0854 | 0.00 | 0.975 |
| *χ^2^*(1) β_C(m)M(m)_=β_C(m)M(a)_ | 165.89 | <0.001 | 120.25 | <0.001 | 119.07 | <0.001 | 107.56 | <0.001 |

**Appendix 4 – Factor Analyses of the Code Misalignment Scale in Study 2b**

We conducted exploratory factor analyses of the items (separately for each vignette, presented in Tables A4.a and A4.b), which yielded two distinct dimensions. One item underperformed in the case of the green technology vignette. In order to maintain comparability across studies and vignettes, we included this item when constructing code misalignment measures. Moreover, confirmatory factor analyses of our intended 2-factor structure yielded acceptable fit (Daycare: CFI = 0.970, RMSEA = 0.104, χ2(19) = 116.462, p < 0.001; Greentech: CFI = 0.927, RMSEA = 0.149, χ2(19) = 220.454, p < 0.001).

| **Table A4.a. Instrument to measure attributions of disagreements to misalignments in causal and moral codes (Daycare vignette) (Study 2b).** | | |
| --- | --- | --- |
| **ITEMS** | **Factor 1** | **Factor 2** |
| They differ in their moral convictions. | **0.96** | -0.11 |
| They have conflicting values. | **0.89** | 0.05 |
| Their views on which course of action is morally appropriate are in contradiction. | **0.79** | 0.07 |
| They are incompatible in terms of their principles. | **0.72** | 0.13 |
| They disagree on what will happen if their respective proposed actions are implemented. | -0.09 | **0.94** |
| They disagree about the consequences of their respective proposed actions. | -0.01 | **0.91** |
| They disagree about the causes and consequences of their proposed actions. | 0.08 | **0.83** |
| Their views on the results of different courses of action are in contradiction. | 0.12 | **0.80** |
| Note. *N* = 463. 'Principal axis factoring' extraction method was used in combination with an ‘oblimin’ rotation. Factor loadings above 0.300 are in bold. | | |

| **Table A4.b. Instrument to measure attributions of disagreements to misalignments in causal and moral codes (Greentech vignette) (Study 2b).** | | | |
| --- | --- | --- | --- |
| **ITEMS** | **Factor 1** | **Factor 2** |  |
| They differ in their moral convictions. | **0.99** | -0.09 |  |
| They have conflicting values. | **0.91** | 0.04 |  |
| Their views on which course of action is morally appropriate are in contradiction. | **0.86** | 0.05 |  |
| They are incompatible in terms of their principles. | **0.82** | 0.07 |  |
| They disagree on what will happen if their respective proposed actions are implemented. | -0.09 | **0.96** |  |
| They disagree about the consequences of their respective proposed actions. | 0.05 | **0.88** |  |
| They disagree about the causes and consequences of their proposed actions. | 0.17 | **0.75** |  |
| Their views on the results of different courses of action are in contradiction. | -0.01 | 0.20 |  |
| Note. *N* = 463. 'Principal axis factoring' extraction method was used in combination with an ‘oblimin’ rotation. Factor loadings above 0.300 are in bold. | | | |

**Appendix 5 – Supplemental Tests of Hypotheses in Study 2b, Using OLS**

In supplemental analysis that we had not registered, we performed two sets of OLS models, one for each vignette to examine vignette-specific effects. As reported in Table A5, code misalignments yield the expected effects for all dependent variables, supporting H1 for each vignette.

To test H2, we compare the coefficient estimates for the dummy variables corresponding to the C(m)M(a) and C(a)M(m) conditions (conditions where only one code is misaligned). As predicted, we find that moral code misalignments had a higher impact on the outcomes than causal code misalignments. These differences are significant for all dependent variables for the green technology vignette. Differences in estimated effects of causal and moral code misalignments fail to reach conventional levels of statistical significance for the daycare vignette for likelihood of conflict resolution, perceived relationship conflict, and negative affect between the parties. However, results remain directionally consistent. Lack of significant findings for the day care vignette may be driven by the small sample size (recall that we had planned and registered the study to perform these analyses on the pooled sample). Moreover, it is also possible that the heightened moralization of issues surrounding green technology differentially enhances the impact of moral (compared to causal) code misalignments.

To test H3, we test the difference between the estimated effect for the C(m)M(m) (both codes in misalignment) condition and the C(m)M(a) (only causal codes misaligned) condition. Tests (presented in the last row in each panel of Table A4) show that misalignment in both codes do have greater effects than misalignment only in causal codes, supporting H3 for both vignettes.

| **Table A5. Study 2 Hypothesis Tests: Estimates from Separate OLS Regressions by Vignette** | | | | | | | | |
| --- | --- | --- | --- | --- | --- | --- | --- | --- |
| **DAYCARE (N=380)** | **Likelihood of conflict resolution** | | **Likelihood of avoiding future engagement** | | **Relationship conflict** | | **Negative affect** | |
|  | **β** | ***p*** | **β** | ***p*** | **β** | **p** | **β** | ***p*** |
| Causal code misalignment only [C(m)M(a)] | 0.582 | <0.001 | 0.675 | <0.001 | 1.147 | <0.001 | 0.643 | <0.001 |
| Moral code misalignment only [C(a)M(m)] | 0.667 | <0.001 | 1.026 | <0.001 | 1.283 | <0.001 | 0.715 | <0.001 |
| Misalignment in both C and M [C(m)M(m)] | 1.549 | <0.001 | 1.698 | <0.001 | 1.888 | <0.001 | 1.392 | <0.001 |
| Intercept | 1.580 | <0.001 | 1.663 | <0.001 | 1.698 | <0.001 | 1.580 | <0.001 |
| Adjusted R2 | 0.322 | | 0.318 | | 0.404 | | 0.251 | |
| **Tests of H2 and H3:** |  |  |  |  |  |  |  |  |
| *F*(1, 376) β_C(m)M(a)_=β_C(a)M(m)_ | 0.5 | 0.482 | 6.94 | <0.001 | 1.24 | 0.265 | 0.32 | 0.573 |
| *F*(1, 376) β_C(m)M(m)_=β_C(m)M(a)_ | 64.91 | <0.001 | 59.62 | <0.001 | 37.69 | <0.001 | 35.26 | <0.001 |
|  |  |  |  |  |  |  |  |  |
| **GREENTECH (N=379)** | **Likelihood of conflict resolution** | | **Likelihood of avoiding future engagement** | | **Relationship conflict** | | **Negative affect** | |
|  | **β** | ***p*** | **β** | ***p*** | **β** | **p** | **β** | ***p*** |
| Causal code misalignment only [C(m)M(a)] | 0.596 | <0.001 | 0.785 | <0.001 | 1.048 | <0.001 | 0.56 | <0.001 |
| Moral code misalignment only [C(a)M(m)] | 1.039 | <0.001 | 1.148 | <0.001 | 1.489 | <0.001 | 0.998 | <0.001 |
| Misalignment in both C and M [C(m)M(m)] | 1.716 | <0.001 | 1.742 | <0.001 | 1.873 | <0.001 | 1.345 | <0.001 |
| Intercept | 1.498 | <0.001 | 1.633 | <0.001 | 1.559 | <0.001 | 1.539 | <0.001 |
| Adjusted R2 | 0.372 | | 0.352 | | 0.404 | | 0.241 | |
| **Tests of H2 and H3:** |  |  |  |  |  |  |  |  |
| *F*(1, 375) β_C(m)M(a)_=β_C(a)M(m)_ | 13.2 | <0.001 | 8.06 | 0.005 | 11.08 | 0.001 | 10.81 | 0.001 |
| *F*(1, 375) β_C(m)M(m)_=β_C(m)M(a)_ | 86.9 | <0.001 | 57.38 | <0.001 | 39.8 | <0.001 | 35.65 | <0.001 |

REFERENCES CITED IN THE APPENDICES:

Dinno, A. (2009). Exploring the sensitivity of Horn’s parallel analysis to the distributional form of random data. *Multivariate behavioral research*, 44(3), 362–388.

Hinkin, T. R. (1998). A brief tutorial on the development of measures for use in survey questionnaires. *Organizational research methods*, 1(1), 104–121.

Horn, J. L. (1965). A rationale and test for the number of factors in factor analysis. *Psychometrika*, 30, 179–185.

Hu, L., & Bentler, P. M. (1999). Cutoff criteria for fit indexes in covariance structure analysis: Conventional criteria versus new alternatives. *Structural Equation Modeling*, 6, 1–55.

Ullman, J. B. (2006). Structural equation modeling. In B. G. Tabachnick & L. S. Fidell (Eds.), *Using multivariate statistics*, (5th ed.; pp. 653–771). Boston: Allyn & Bacon.
